# Hybrid Slime Mold and Arithmetic Optimization Algorithm with Random Center Learning and Restart Mutation

**DOI:** 10.3390/biomimetics8050396

**Published:** 2023-08-28

**Authors:** Hongmin Chen, Zhuo Wang, Heming Jia, Xindong Zhou, Laith Abualigah

**Affiliations:** 1 Department of Information Engineering, Sanming University, Sanming 365004, China; chm@fjsmu.edu.cn (H.C.); 20200862240@fjsmu.edu.cn (Z.W.); 20190852137@fjsmu.edu.cn (X.Z.); 2 Prince Hussein Bin Abdullah College for Information Technology, Al Al-Bayt University, Mafraq 25113, Jordan; aligah.2020@gmail.com; 3 Department of Electrical and Computer Engineering, Lebanese American University, Byblos 13-5053, Lebanon; 4 Hourani Center for Applied Scientific Research, Al-Ahliyya Amman University, Amman 19328, Jordan; 5 MEU Research Unit, Middle East University, Amman 11831, Jordan; 6 Applied Science Research Center, Applied Science Private University, Amman 11931, Jordan; 7 School of Computer Sciences, Universiti Sains Malaysia, Gelugor 11800, Malaysia

**Keywords:** slime mold algorithm, arithmetic optimization algorithm, random center solution strategy, restart strategy, mutation strategy

## Abstract

The slime mold algorithm (SMA) and the arithmetic optimization algorithm (AOA) are two novel meta-heuristic optimization algorithms. Among them, the slime mold algorithm has a strong global search ability. Still, the oscillation effect in the later iteration stage is weak, making it difficult to find the optimal position in complex functions. The arithmetic optimization algorithm utilizes multiplication and division operators for position updates, which have strong randomness and good convergence ability. For the above, this paper integrates the two algorithms and adds a random central solution strategy, a mutation strategy, and a restart strategy. A hybrid slime mold and arithmetic optimization algorithm with random center learning and restart mutation (RCLSMAOA) is proposed. The improved algorithm retains the position update formula of the slime mold algorithm in the global exploration section. It replaces the convergence stage of the slime mold algorithm with the multiplication and division algorithm in the local exploitation stage. At the same time, the stochastic center learning strategy is adopted to improve the global search efficiency and the diversity of the algorithm population. In addition, the restart strategy and mutation strategy are also used to improve the convergence accuracy of the algorithm and enhance the later optimization ability. In comparison experiments, different kinds of test functions are used to test the specific performance of the improvement algorithm. We determine the final performance of the algorithm by analyzing experimental data and convergence images, using the Wilcoxon rank sum test and Friedman test. The experimental results show that the improvement algorithm, which combines the slime mold algorithm and arithmetic optimization algorithm, is effective. Finally, the specific performance of the improvement algorithm on practical engineering problems was evaluated.

## 1. Introduction

In the past decade, the exploitation and application of optimization models have begun to receive attention from mathematicians and engineers. In recent years, with the continuous exploitation of computer technology, more and more optimization problems have attracted people’s attention. The unconstrained optimization problem is currently a research hotspot. The complexity of these problems is gradually increasing, and they have characteristics such as being large-scale, multimodal, and nonlinear [1]. The meta-heuristic algorithm has become an excellent tool and has been recognized by people because it is simple, easy to implement, does not require gradient information, and can avoid local optimization [2]. This is because the meta-heuristic algorithm treats the problem as a black box model and only needs to input the problem to obtain the output of the problem. Researchers develop meta-heuristic algorithms by simulating various natural phenomena and biological habits. Meta-heuristic algorithms can effectively handle real-life optimization problems because they possess valuable randomness that allows them to bypass local optima and have stronger search capabilities than traditional optimization algorithms. These valuable characteristics of the meta-heuristic algorithm make it very smooth when dealing with application problems. Examples include neural networks [3], clustering [4], engineering [5], and scheduling problems [6].

The main problem is whether a meta-heuristic algorithm can be used to solve most of the problems. No Free Lunch (NFL) [7] explains that when an algorithm can provide a good solution to a particular problem, it is not guaranteed a good result on other problems. NFL’s law motivates researchers to enhance their ability to solve new problems by improving currently known algorithms. For example, Chen et al. were inspired by the lifestyle of beluga whales and developed an IBWO [8] that improved the algorithm’s global optimization ability; Wen et al. enhanced the global optimization capability of the algorithm by using a new host-switching mechanism [9]; Wu et al. improved the sand cat’s wandering strategy and applied it to engineering problems [10].

AOA [11] is a meta-heuristic optimization algorithm designed based on the four mixed operations proposed by Abualigah et al. in 2021. The algorithm uses multiplication and division operations in mathematics to improve the global dispersion of position updates and addition and subtraction operations to improve the accuracy of position updates in local areas. However, AOA still faces problems such as slow convergence in complex environments. It needs further improvement and perfecting to adapt to more complex optimization problems. Recently, many researchers have made improvements to the AOA, including adaptive parallel arithmetic optimization algorithm (AAOA) [12], dynamic arithmetic optimization algorithm (DAOA) [13], and chaotic arithmetic optimization algorithm (CAOA) [14].

SMA [15] is a new swarm intelligence optimization algorithm proposed by Li et al. in 2020 to simulate slime mold’s behavior and morphological changes in the foraging process. Its inspiration comes from simulating the foraging behavior and morphological changes of physarum polycephalum and using the weight change to simulate the positive feedback and negative feedback processes generated by slime molds in the foraging process, thus generating three stages of foraging patterns. The SMA has strong global exploration ability, certain convergence accuracy, and good stability, but in the later iteration stage, the oscillation effect is weak, and it is easy to fall into local optima. The contraction mechanism is not strong, resulting in a slower convergence speed. At present, researchers have improved and widely used this algorithm. Kouadri et al. [16] applied the algorithm to solve the problem of minimizing fuel costs and losses in exploration generators. Zhao et al. [17] proposed mixing SMA and HHO algorithms, utilizing multiple composite selection mechanisms to improve the algorithm’s selectivity and randomness, the randomness of individual position updates, and the efficiency of algorithm solving.

Based on the advantages and disadvantages of SMA and AOA, this article aims to create a more effective optimization algorithm by combining the two algorithms. To further enhance its performance, a random center solution strategy is introduced. This strategy uses random center learning to expand the exploration range of individual populations, enrich population diversity, and can effectively control the balance between exploration and exploitation. Both mutation strategy and restart strategy were used. The mutation strategy is a local adaptive mutation method that improves the algorithm’s global search ability and performs well in high-dimensional spaces. The restart strategy can help poorer individuals jump to other positions and is usually used to jump out of local optima. The proposed mixed optimization algorithm, hybrid slime mold and arithmetic optimization algorithm with random center learning and restart mutation (RCLSMAOA), incorporates the best features of both SMA and AOA, making it more effective in exploring the search space and enabling it to effectively solve corresponding engineering problems. The algorithm focuses on improving four key aspects:

(1) In the exploration and exploitation stage, SMA and AOA should be organically combined to improve the exploration and exploitation capabilities comprehensively;
(2) Innovatively propose a random center strategy, which improves the early convergence speed of the algorithm and effectively maintains a balance between exploration and development while enhancing the diversity of the population;
(3) The introduction of the mutation strategy and restart strategy enhances the ability to solve complex problems while also enhancing the algorithm’s ability to jump out of local optima. By comparing 23 benchmark test functions with different dimensions with the CEC2020 test function, it is proven that the algorithm has significant effectiveness;
(4) Five engineering problems were used simultaneously to verify the feasibility of RCSMAOA on practical engineering problems.

The second part of this article introduces the relevant work. SMA and AOA were introduced in the third and fourth parts, respectively. In the fifth part, we described the added strategies; SCLS, MS, and RS explained the implementation process of the algorithm and provided pseudocode and flowchart. The sixth part is the analysis of time complexity. The seventh part is the experimental analysis of the specific performance of RCLSMAOA. The eighth part is the application of RCLSMAOA in specific engineering problems. The ninth part summarizes the contributions of this article and introduces the next research directions. If you need the code in our article, you can find it through the following link: https://github.com/Mars02w/RCLSMAOA, accessed on 20 August 2023.

## 2. Related Works

In recent years, meta-heuristic algorithms can be divided into the following four categories based on their inspiration sources: (1) physics-based methods, whose inspiration comes from physical rules in the universe. Specific algorithms include the black hole algorithm (BH) [18], the gravity search algorithm (GSA) [19], and the most famous simulated annealing algorithm (SA) [20]. (2) Evolution-based algorithms inspired by the laws of biological evolution. Researchers have linked natural and artificial evolution to create many excellent algorithms. Examples include genetic algorithm (GA) [21], genetic programming (GP) [22], and differential evolution (DE) [23]. (3) Group-based algorithms focus on modeling observations of animals and other living organisms. The most famous is particle swarm optimization (PSO) [24], which simulates the behavior of birds and fish. Ant Colony Algorithm (ACO) [25] simulates the behavior of ants searching for food sources. The foraging behavior of slime molds inspires the slime mold optimization algorithm (SMA) [15]. (4) Human behavior habits and ideas inspire human-based algorithms in social life. A well-known one is the teaching–learning-based optimizer (TLBO) [26], inspired by the interaction between teachers and students. There are also training-based optimizers (DTBO) [27] and internal search algorithms (ISA) [28]. In recent years, many excellent algorithms have still been proposed by researchers, such as the monarch Butterfly optimization (MBO) [29], more search algorithm (MSA) [30], hunger games search (HGS) [31], Runge Kutta method (RUN) [32], colony prediction algorithm (CPA) [33], weighted mean of vectors (INFO) [34], Harris hawks optimization (HHO) [35], and prime optimization algorithm (RIME) [36].

In addition, studying hybrid optimization algorithms is also a new trend at present. In recent years, researchers have increasingly conducted mixed research on algorithms. For example, Alam Zeb et al. [37] mixed a genetic algorithm with a simulated annealing algorithm, and the powerful exploration ability of the genetic algorithm compensated for the lack of exploration in the simulated annealing algorithm, enhancing the actual optimization performance of the algorithm. Chen et al. [38] fused a particle swarm optimization algorithm with a simulated annealing algorithm and applied it to magnetic anomaly detection, achieving success. Hybrid optimization algorithms also have the ability to solve optimal power flow problems [39]. Tumari et al. studied a variant of the ocean predator algorithm for adjusting the fractional order proportional integral derivative controller of the automatic voltage regulator system [40]. Wang et al. [41] added an angle modulation mechanism to a dragonfly algorithm to enable it to work in two-dimensional space and have good performance. Devaraj A et al. [42] used a combination of fireflies and improved multi-objective particle swarm optimization (IMPSO) technology to improve load balancing capabilities in cloud computing environments and the results showed success. Jui et al. [43] hybridized the average multi-verse optimizer and sine cosine algorithm, demonstrating good potential in solving continuous-time Hammerstein system problems.

## 3. Slime Mold Algorithm

The SMA is a meta-heuristic optimization algorithm that simulates the foraging behavior of slime molds. This algorithm reflects slime molds’ oscillation and contraction characteristics during the foraging process. The organic matter in slime molds first secretes enzymes when searching for food, and then during migration, the front end extends into a fan shape and uses a venous network for foraging. Due to their unique morphology and characteristics, they can simultaneously utilize multiple pathways to form a connected venous network to obtain food. In addition, slime molds will also search for food in other unknown areas.

When the slime mold vein is close to food according to the smell in the air, the higher the food concentration, the stronger the propagation wave generated by the biological oscillator in its body, which increases the flow of cytoplasm in the vein. The faster the flow of cytoplasm, the thicker the slime mold vein tube, which causes the position update of the slime mold. The position update formula is:

(1) $$X(t+1)=\left\{\begin{array}{l} X_{b}(t)+vb\times (W\times X_{rand1}(t)-X_{rand2}(t)),r_{1}<p\\ \;\;\;\;\;vc\times X(t)\;\;\;\;\;\;\;\;\;\;\;\;,r_{1}\geq p \end{array}\right.$$

where *X_b_(t)* represents the current position of the individual with the optimal fitness, *vb* is a parameter between [-*a*,*a*], *W* represents the weight coefficients of the slime mold, *X_rand1_*(*t*) and *X_rand2_*(*t*) represent the positions of two randomly selected individuals, *r_1_* represents the random number in the interval [0, 1], *vc* is the parameter that linearly decreases from 1 to 0, and *t* is the current number of iterations.

The updating formula of control parameters *a*, *p*, and weight coefficient *W* is as follows:

(2) $$a=\arctan h\left(-\left(\frac{t}{T}\right)+1\right)$$

(3) $$p=\tanh\left|S(i)-DF\right|$$

(4) $$W(SIndex)=\left\{\begin{array}{l} 1+r_{2}\times \log\left(\frac{bF-S(i)}{bF-wF}+1\right),condition\\ 1-r_{2}\times \log\left(\frac{bF-S(i)}{bF-wF}+1\right),others \end{array}\right.$$

(5) SIndex(i)=sort(N)

The parameters are calculated based on the current individual’s fitness and optimal values, where *i* = 1,2,…, *N. N* represents the number of populations, *S(i)* represents the fitness value of the *i*th slime mold individual, and *DF* represents the optimal fitness obtained in the current iteration process. *T* represents the maximum number of iterations, and *r*_2_ is a random number within the [0, 1] interval. *condition* are the individuals whose fitness is in the top half of the population, and *others* are the remaining individuals. *bF* represents the best fitness value obtained during the current iteration, and *wF* represents the worst fitness value obtained during the current iteration. *SIndex(i)* indicates the fitness value sequence.

Even if slime molds find a food source, they will separate some individuals to explore other unknown areas to find a higher-quality food source. At this point, the formula for the slime mold update position is as follows:

(6) $$X(t+1)=\left\{\begin{array}{l} rand\times (UB-LB)+LB,rand<z\\ X_{b}(t)+vb\times (W\times X_{rand1}(t)-X_{rand2}(t)),r_{1}<p\\ vc\times X(t),r_{1}\geq p \end{array}\right.$$

Among them, *rand* is a random number within the [0, 1] interval, *UB* and *LB* represent upper and lower bounds, and *z* represents a custom parameter (with a value of 0.03).

## 4. Arithmetic Optimization Algorithm

AOA is a meta-heuristic optimization algorithm exploring and exploiting mechanisms through arithmetic operators in mathematics. The exploration stage refers to using multiplication (M) and division (D) strategies for extensive coverage and search space, improving the dispersion of solutions to avoid local optima. The exploitation stage is to improve the accuracy of the solutions obtained in the exploration stage through the addition (A) strategy and the subtraction (S) strategy, that is, to enhance the local optimization ability.

### 4.1. Mathematical Optimization Acceleration Function

Before exploration and exploitation, AOA generates a math optimizer accelerated (MOA) to select the search phase. When *r*_1_ > MOA (t), the exploration phase is carried out by executing (D) or (M); when *r*_1_ ≤ MOA (t), the exploitation phase is carried out by executing (A) or (S); *r*_1_ is a random number from 0 to 1.

(7) $$MOA(t)=Min+t\times \left(\frac{Max-Min}{T}\right)$$

where Min and Max represent the minimum and maximum values of the optimization acceleration function (MOA).

### 4.2. Exploration Phase

AOA looks at different parts of the search space (global optimization) using two main search methods (multiplication (M) search strategy and division (D) search strategy). It updates its position using this formula:

(8) $$X_{i,j}(t+1)=\left\{\begin{array}{l} X_{b,j}(t)÷(MOP+\varepsilon )\times ((UB_{j}-LB_{j})\times \mu +LB_{j}),r_{2}<0.5\\ X_{b,j}(t)\times MOP\times ((UB_{j}-LB_{j})\times \mu +LB_{j})\;\;\;\;\;\;,others \end{array}\right.$$

where r_2_ ∈ [0, 1], X_i,j_(t + 1) is the position of the ith individual on the jth dimension during the next iteration. X_b,j_(t) is the location of the best solution for the current fitness. ɛ is a very small number, where UB_j_ and LB_j_ represent the upper and lower limits of the jth position, respectively. µ is the control parameter for adjusting the search process, which is 0.499.

(9) $$MOP(t)=1-\frac{t^{1/\alpha }}{T^{1/\alpha }}$$

The Mathematical Optimizer Probability (MOP) is a coefficient, α is a sensitive parameter that defines the exploitation accuracy during the iteration process, which is fixed at 5.

### 4.3. Exploitation Phase

AOA utilizes operators (subtraction (S) and addition (A)) to deeply explore search areas in several dense areas and conducts local optimization based on two main exploitation strategies. The location update formula is as follows:

(10) $$X_{i,j}(t+1)=\left\{\begin{array}{l} X_{b,j}(t)-MOP\times ((UB_{j}-LB_{j})\times \mu +LB_{j}),r_{3}<0.5\\ X_{b,j}(t)+MOP\times ((UB_{j}-LB_{j})\times \mu +LB_{j}),others \end{array}\right.$$

Among them, r_3_ is a random number between 0 and 1.

## 5. Hybrid Improvement Strategy

### 5.1. Stochastic Center Learning Strategy (SCLS)

The random center learning strategy is a newly proposed optimization mechanism in this paper. In nature, group animals such as wolf packs and whale packs often surround their food in the middle through group cooperation and then engage in predation. In this regard, this article proposes a central learning strategy whose core idea is to generate a central solution based on upper and lower bounds during the process of searching for the optimal value of the population, comparing it with the target fitness value of the existing optimal solution, and selecting the optimal one for the next iteration. Definition of the central solution: if there is a point X in the n-dimensional coordinate system, then the central solution is calculated as follows:

(11) $$X_{c}=(LB+UB)/2$$

**Figure 1:**
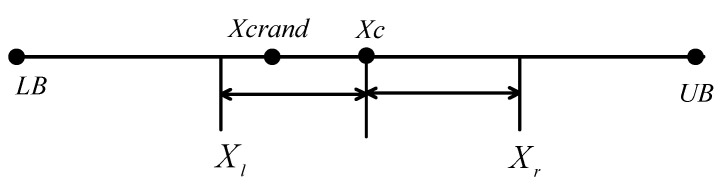
Central solution and random central learning solution.

Among them, X_c_ is the central solution. Due to the lack of randomness in the central solution. In order to further improve the ability of the population to find the globally optimal solution (as shown in Figure 1), this paper proposes an improved random center learning strategy, which is calculated as follows:

(12) $$X_{crand}=\left\{\begin{array}{cc} X_{c}+(X_{r}-X_{c})\cdot r_{1}, & rand()<0.5\\ X_{c}+(X_{c}-X_{l})\cdot r_{2}, & rand()>0.5 \end{array}\right.$$

where *X_crand_* represents the random central solution, *X_r_* and *X_l_* represent the object to be learned, and *r*_1_, *r*_2_, and *rand* are random numbers between 0 and 1. In order to reflect the randomness and symmetry of the value of random center learning, the threshold value of rand () is 0.5. The schematic diagram of the central solution and random central learning is shown in Figure 1.

Figure 1 shows that any position in the interval [*X_l_*, *X_r_*] may have a random central solution *X_crand_*, with *X_c_* being the central solution.

### 5.2. Mutation Strategy (MS)

Mutation strategy refers to a composite mutation strategy based on multiple mutation operators in the mutation strategy [44]. It generates three candidate positions *V_i_*_1_, *V_i_*_2_, and *V_i_*_3_, for the ith position by executing Equations (13)–(15) in parallel. The formula is as follows:

(13) $$V_{i1,j}=\left\{\begin{array}{l} X_{r1,j}+F_{1}\times \left(X_{r2,j}-X_{r3,j}\right),\;if\;rand()<C_{r1}\;or\;j=j_{rand}\\ \;\;\;\;X_{i,j}\;\;\;\;\;,others \end{array}\right.$$

Among them, *X_rk, j_* represents the jth dimension of the *r_k_* solution (the same below), *r*_1_, *r*_2_, and *r*_3_ are different integers in the range [1, N], *j_rand_* represents integers in the interval [1, D], *F_1_* represents a proportional coefficient equal to 1.0, and *C_r1_* represents a crossover rate set to 0.1.

(14) $$V_{i2,j}=\left\{\begin{array}{l} X_{r4,j}+F_{2}\times \left(X_{r5,j}-X_{r6,j}\right)+F_{2}\times \left(X_{r7,j}-X_{r8,j}\right),\;if\;rand()<C_{r2}\;or\;j=j_{rand}\\ \;\;\;\;\;\;\;\;X_{i,j}\;\;\;\;\;\;\;\;\;,others \end{array}\right.$$

*r*_4_, *r*_5_, *r*_6_, r_7_, and *r*_8_ are distinct integers in the range [1, N]. *F*_2_ represents a proportional coefficient equal to 0.8, and *C_r2_* represents a crossover rate equal to 0.2.

(15) $$V_{i3,j}=\left\{\begin{array}{l} X_{i,j}+rand()\times \left(X_{r9,j}-X_{i,j}\right)+F_{3}\times \left(X_{r10,j}-X_{r11,j}\right),\;if\;rand()<C_{r3}\;or\;j=j_{rand}\\ \;\;\;\;\;\;\;\;X_{i,j}\;\;\;\;\;\;\;\;\;,others \end{array}\right.$$

*r*_9_, *r*_10_, and *r*_11_ are distinct integers in the range [1, N]. *F*_3_ represents a proportional coefficient equal to 1.0, and *C_r3_* represents a crossover rate of 0.9.

After generating three candidate positions *V_i_*_1_, *V_i_*_2_, and *V_i_*_3_, first correct them based on the upper and lower boundaries. Then, select the candidate solution *V_i_* with the best fitness from *V_i_*_1_, *V_i_*_2_, and *V_i_*_3_ to update the position of the *i*th solution using Formula (16), which is called a greedy selection strategy, as shown below.

(16) $$X_{i}=\left\{\begin{array}{l} V_{i}\;,\;if\;f(V_{i})<f(X_{i})\\ X_{i},\;otherwise \end{array}\right.$$

*V*_i_ represents the modified best candidate solution, and *X_i_* represents the ith position.

### 5.3. Restart Strategy (RS)

When the mucus cannot find food at this location for a long time, it means that the nutrients in the area are no longer sufficient to support the continued survival of the slime molds, so the slime molds in the area need to be relocated. The restart scheme [9] can help poorer individuals transition from a local optimal state to other positions, so we use a restart strategy here to change the position of poorer individuals. In this strategy, we use the trial vector *trial(i)* to record the number of times the position has not improved. If the fitness value corresponding to the position in the search does not improve, the test vector *trial(i)* increases by 1. Otherwise, *trial(i)* is reset to 0. If the test vector is not less than the predetermined limit, a better vector will be selected from the test vectors *T*_1_ and *T*_2_ to replace the position of the ith.

(17) $$T_{1,j}=LB_{j}+rand()\times \left(UB_{j}-LB_{j}\right)$$

(18) $$T_{2,j}=rand()\times \left(UB_{j}+LB_{j}\right)-X_{i,j}$$

(19) $$T_{2,j}=LB_{j}+rand()\times \left(UB_{j}-LB_{j}\right)if\;T_{2,j}\geq UB_{j}||T_{2,j}\leq LB_{j}$$

where *T*_1*,j*_ represents the jth dimension position in position *T*_1_, *T*_2*,j*_ represents the jth position in position *T*_2_, *UB_j_* and *LB_j_* are the upper and lower boundaries of the jth dimension, respectively, and *rand*() represents the random floating-point arithmetic in the region [0, 1]. If *T*_2*,j*_ exceeds the upper boundary *UB_j_* or lower boundary *LB_j_* in the jth dimension of the position, it will be replaced by Equations (18) and (19), and the test vector test *trial (i)* will be reset to zero. This article sets this limit to log t. If the restrictions are smaller in the early stages, they will help enhance the global performance of the algorithm. If the limit is larger in the later stage, it can prevent the algorithm from moving away from the optimal solution.

### 5.4. A Hybrid Optimization Algorithm of Slime Mold and Arithmetic Based on Random Center Learning and Restart Mutation

As mentioned above, when exploring unknown food sources, slime molds in SMA update their positions based on the synergistic effect of *V_B_* and *Vc*. The oscillation effect of *V_B_* increases the possibility of global exploration. When the random number *z* is less than, slime molds are initialized. At the end of the iteration, the *V_B_* oscillation effect is weakened, which makes the algorithm easily fall into the local optimum. *Vc* is a linearly decreasing parameter from 1 to 0, and the search mechanism is single and weak, making it difficult for the algorithm to jump out of local optima. In AOA, position updating is carried out according to the higher distribution of the division operator, and contraction is realized according to the addition and subtraction operators. The probability MOP of the mathematical optimizer changes according to the optimal position to improve the search breadth of exploration and increase the ability of the algorithm to jump out of the local optimum, but it will inevitably fall into the local optimum in the later iteration. The random center learning updates the random position according to the general characteristics of the optimal solution, which will improve the algorithm’s convergence rate in the early exploration stage. CMS introduces multiple candidate solutions and compares them with existing solutions. In RS, the number of times the position has not been improved is recorded using the experimental vector trial (i). When the given threshold is exceeded, it is preliminarily determined that the algorithm is trapped in a local optimum, and a new position update formula is given to prevent the algorithm from jumping out of the local optimum.

Therefore, in this paper, we abandon the weak mechanism in the exploration stage of SMA and add the multiplication and division operator in AOA to expand the scope of exploration. The mutation and restart strategies are introduced to improve the ability to jump out of the local optimal in the late iteration. Given the relatively slow convergence rate of the algorithm in the exploration stage, a random center learning strategy with characteristic solutions is added.

**Figure 2:**
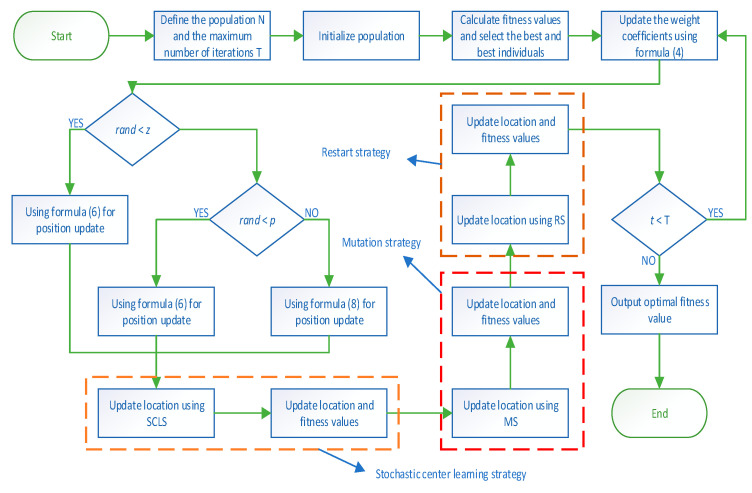
Flow chart.

To sum up, the hybrid slime mold and arithmetic optimization algorithm with random center learning and restart mutation (RCLSMAOA) proposed in this paper, which integrates stochastic center learning, has advantages and balance in the exploration and exploitation stage, local optimization, and global optimization. Pseudocode as shown in Algorithm 1. The flowchart is shown in Figure 2.
**Algorithm 1** The pseudocode of the RCLSMAOAInitialization parameters *T*, *Tmax*, *ub*, *lb*, *N*, *dim*, *w*.Initialize population *X* according to Equation (1).**While** T ≤ Tmax   Calculate fitness values and select the best individual and optimal location.   Update *w* using Formula (4)      **For** *i* = 1:*N*       Update the value of parameter *a W S* using Formulas (2), (4), and (5)      **If** *rand* < *z*       Update the population position using Formula (6)      **Else**
       Update *vb*, *vc*, and *p.*       **If** *r_1_* < *p*         Update the population position using Formula (6)       **Else**
         Update the value of parameter mop using Formula (9)         **If** *r_2_* < 0.5            Update the population position using Formula (8)         **Else**
            Update the population position using Formula (8)         **End If**
       **End If**
     **End If**
     **For** *i* = 1:*N*         Update population position using SCLS     **End For**
     **For** *i* = 1:*N*         Update population position using MS     **End For**
     Update population position using RS   Find the current best solution   *t* = *t* + 1**End While**Output the best solution.

## 6. Time Complexity Analysis

In the RCLSMAOA, if the number of populations is *N*, the dimension is dim, and the maximum number of iterations is *T*. The time complexity of the population initialization phase is O(N × Dim). During iteration, the location of vb mechanism and AOA multiplication and division operator in SMA is updated, and the time complexity of the random central solution strategy and mutation strategy is O(3 × N × Dim × T). The time complexity of updating the convergence curve is O(1). It is worth mentioning that RS is rarely used from a general perspective, so it can be ignored and not remembered. In conclusion, the time complexity of the RCLSMAOA is O(N × Dim × (3T + 1)).

## 7. Experimental Part

All the experiments in this paper are completed on the computer with the 11th Gen Intel(R) Core(TM) i7-11700 processor with a primary frequency of 2.50 GHz, 16 GB memory, and an operating system of 64-bit Windows 11 using matlab2021a. In order to check the performance of the RCLSMAOA, 23 standard reference functions and CEC2020 reference functions are used to check the algorithm’s performance. In order to have a more comprehensive understanding of the actual performance of the RCLSMAOA, we choose different algorithms to compare. These include AOA and SMA, as well as the famous remora optimization algorithm (ROA) [37], sine cosine algorithm (SCA) [38], and whale optimization algorithm (WOA) [39]. In addition, we have added two improved algorithms: the whale and moth flame optimization algorithms and the average multi-verse optimizer and sine cosine algorithm. The parameter settings for these algorithms are shown in Table 1.

### 7.1. Experiments on the 23 Standard Benchmark Functions

**Figure 3:**
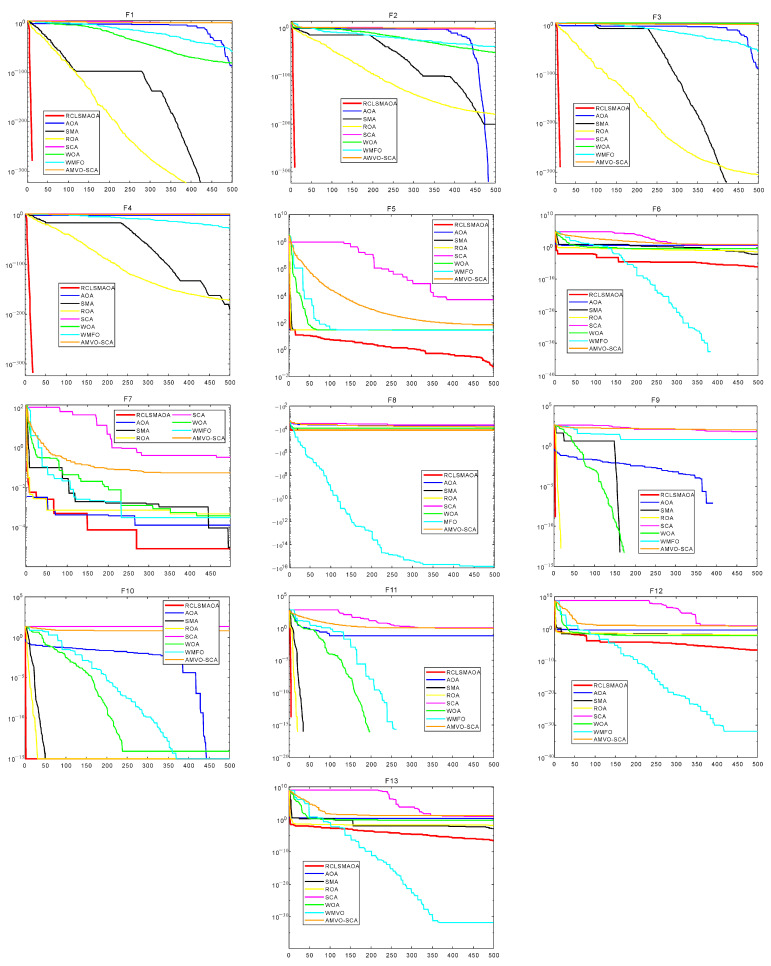
F23 function images with dim = 30 (F1–F13).

In this section, we selected 23 benchmark functions to test RCLSMAOA’s performance [10]. The 23 functions consist of 7 single-mode functions, six multimodal functions, and ten fixed multimodal functions. Fn(x) represents the specific mathematical expression of the reference function, dim is the experimental dimension of the reference function, the range is the search space of the reference function, and Fmin is the theoretical optimal value of the corresponding reference function. See Figure 3 for the image of the specific function. In this experiment, we set the population size N = 30, spatial dimension = 30/500, and the maximum number of iterations T = 500. RCLSMAOA and other comparison algorithms were run 30 times to obtain each algorithm’s best fitness, average fitness, and standard deviation after 30 times of independent running.

The specific experimental table is shown in Table 2, Table 3 and Table 4. We can see that on F1–F7, RCLSMAOA obtained the optimal values among three data items, including the optimal fitness value. We observed that the AOA performed well on F2, whereas the SMA performed well on F1 and F3. The RCLSMAOA inherits its advantages in single-mode functions. On the 500 dimensional scales, the RCLSMAOA still performs well. This is because mutation strategies can perform local mutations and increase global exploration capabilities.

Similarly, we observed multimodal functions such as F8–F13. On F8, the optimal fitness value and the average value of RCLSMAOA reached the optimal value, and the standard deviation was slightly lower than that of SMA. Functions such as F9–F11 are relatively simple, giving most optimization algorithms good results. The performance of RCLSMAOA in the F12–13 function is also satisfactory. We observed that the performance of the RCLSMAOA will not be affected by changes in dimensions, and its performance remains stable. Functions such as F14–23 are fixed multimodal functions, which are relatively simple. In our experiment, it is not difficult to see that the performance of RCLSMAOA is still the best among the comparison algorithms. Although fixed multimodal functions are relatively simple, their performance in verifying algorithm performance is still reliable. The above analysis indicates that RCLSMAOA, which integrates SMA and AOA, performs better than SMA and AOA.

**Figure 4:**
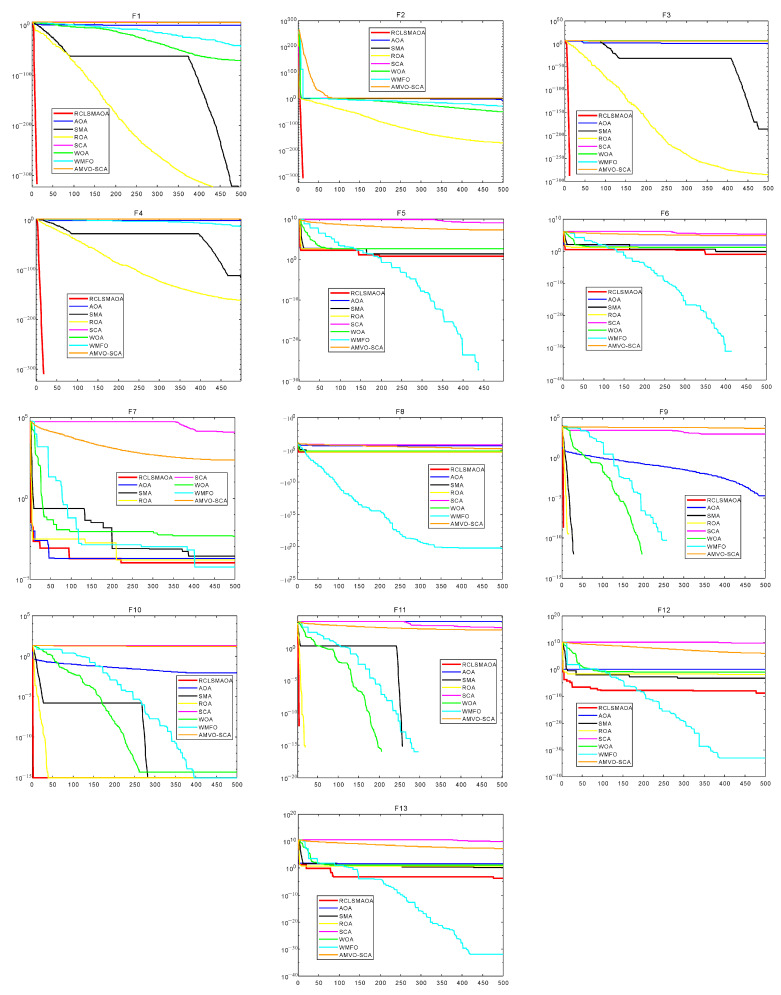
F23 function images with dim = 500 (F1–F13).

**Figure 5:**
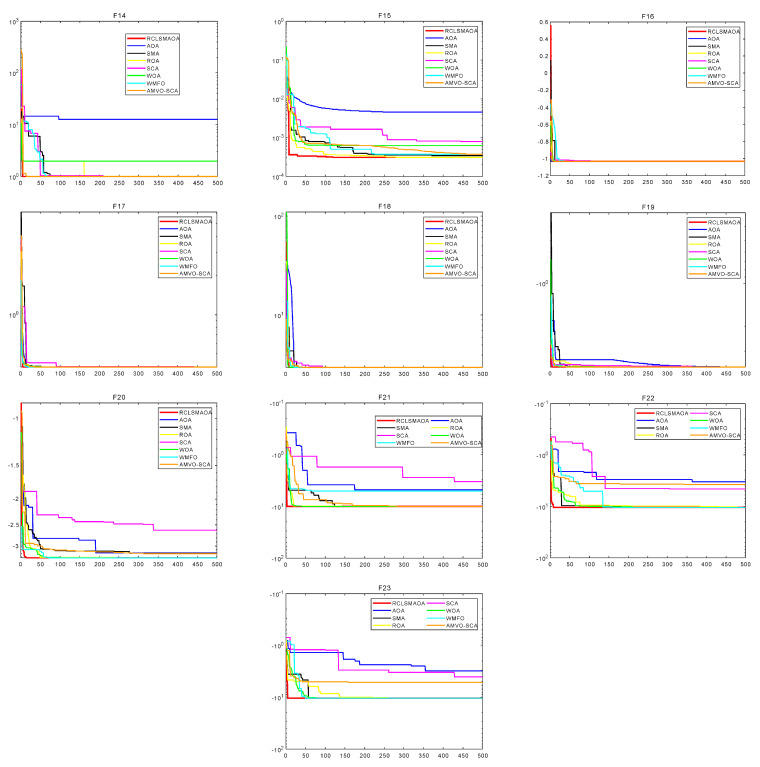
F23 function images (F14–F23).

The data cannot intuitively understand the actual performance of the algorithm so we will show the convergence curves of each algorithm on F23 function images. The function image is shown in Figure 3, Figure 4 and Figure 5. From the image, we can see that in F1–F4, the RCLSMAOA has a fast convergence rate and high convergence precision, which SMA and AOA do not possess. This is due to the random center learning strategy, which expands the algorithm’s search range and enhances the convergence rate. For F5–6, RCLSMAOA can find a good position at the beginning of the iteration and then slowly converge to find the optimal position. Except for the WMFO algorithm, other algorithms stagnate in the early stages of the algorithm. For F7, the optimization ability of this algorithm is also stronger than other algorithms, because the existence of a restart strategy enables the algorithm to continuously jump out of local optima. On F8, the performance of RCLSMAOA is not as good as the WMFO algorithm, but stronger than other algorithms. F9–F11 is relatively simple and easy to find the optimal fitness value. RCLSMAOA algorithm is also the algorithm with the fastest rate of convergence. For F12–F13, the RCLSMAOA performs well and can also converge when other algorithms fall into local optima. F14–23 is a relatively simple function, but it can also play a role in verifying algorithm performance. On these functions, RCLSMAOA also always finds the optimal value the fastest. In summary, the RCLSMAOA applies to F23 functions.

### 7.2. Experiments on the CEC2020 Benchmark Function

Using F23 functions for validation is not sufficient. We have added the CEC2020 test function [9] to verify this. In this experiment, we set the variables as N = 30, T = 500, and dim = 10. The comparison algorithm remains unchanged. The results of 30 runs of RCLSMAOA and other algorithms are shown in Table 5.

CEC2020 has four class functions: unimodal function CEC01, basic multimodal function CEC02−4, mixed function CEC05-6, and combination function CEC06-10. In unimodal functions, the best one is RCLSMAOA, followed by SMA. This is because the RCLSMAOA integrates the SMA and adds mutation strategies to enhance its exploitation capabilities, enabling it to find better locations. The RCLSMAOA always performs stably based on multimodal functions and can find better values. This is based on the fact that the random center-solving strategy can effectively maintain a balance between exploration and exploitation in RCLSMAOA. Combined with the search strategy in SMA, the RCLSMAOA performs well on the basic multimodal functions. Mixed and combined functions are relatively difficult and complex and can easily trap functions into local optima. For this reason, we introduce a restart strategy to enable the RCLSMAOA to jump out of local optima. From the implementation results, the RCLSMAOA performs well and is not troubled by local optima.

**Figure 6:**
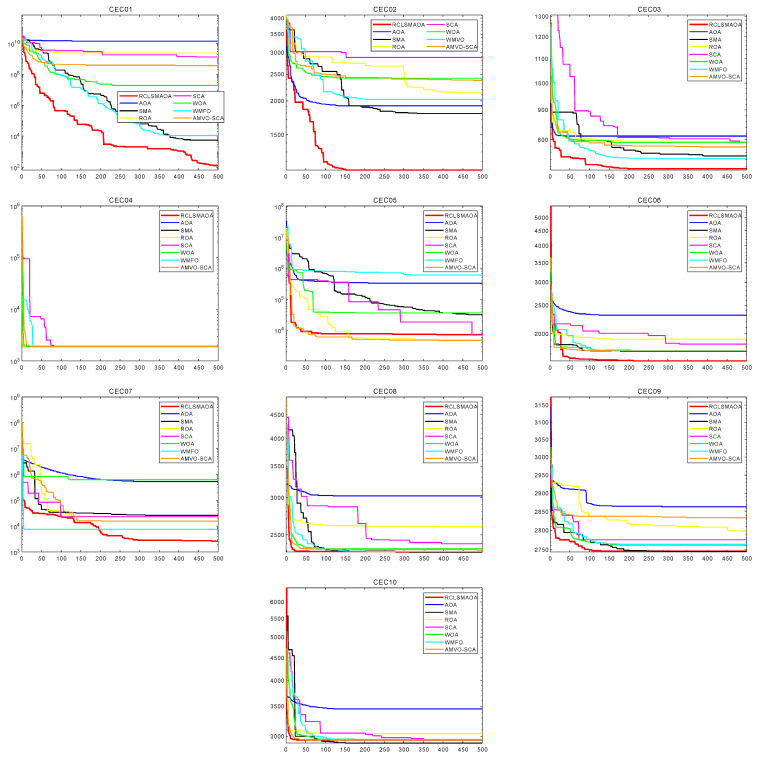
Specific function image of CEC2020 test function.

To test the actual performance of the algorithm more clearly, we selected the specific convergence curves of the RCLSMAOA and other comparative algorithms, as shown in Figure 5. The convergence curves of the RLCSMAOA are mainly divided into two types. One is mainly reflected in the single-mode function. The RCLSMAOA can converge towards the optimal value and finally find the optimal value. This is because the RCLSMAOA integrates the position update formula from the SMA and uses a mutation strategy to improve it. Another is mainly reflected in the complex combination function and mixed function. To test the actual performance of the algorithm more clearly, we selected the specific convergence curves of the RCLSMAOA and other comparative algorithms, as shown in Figure 6. The convergence curves of the RLCSMAOA are mainly divided into two types. One is mainly reflected in the single-mode function. The RCLSMAOA can converge toward the optimal value and finally find the optimal value. This is because the RCLSMAOA integrates the position update formula from the SMA and uses a mutation strategy to improve it. Another is mainly reflected in the complex combination function and mixed function. For complex functions, RCLSMAOA shows a very fast convergence rate at the early stage of iteration. This is because RCLSMAOA uses the multiplication and division operator in the AOA, which allows the RCLSMAOA to find the optimal value very quickly.

### 7.3. Analysis of Wilcoxon Rank Sum Test Results and Friedman Test

Wilcoxon Rank Sum Test Results is a non-parametric detection test method that does not require any assumptions to be made about the data. Therefore, it applies to various types of data, including discrete, continuous, normal, and non-normal distribution. In this experiment, it was used to test whether two samples have differences. The experimental result of this experiment is p, when p is less than 5%. We believe there is a significant difference in the experimental results. Because the RCLSMAOA cannot compare with itself, we will not list the specific *p*-values of RCLSMAOA. Therefore, this article takes eight algorithms as samples; each algorithm independently solves 30 times and sets the population size N = 30. Among them, the dimensions selected for testing 23 standard test functions are 30 dimensions, and CEC2020 is ten dimensions.

Table 6 shows the experimental results of thirty experiments conducted on 23 standard test functions. Table 6 shows the experimental results of 30 experiments conducted on 23 standard test functions. For F1–F4, because the RCLSMAOA is a hybrid form of SMA, they are not distinguished in some functions. For F7–F11, these functions are simple, and most algorithms can achieve good results on them.

Table 7 shows the experimental results of thirty experiments conducted on the CEC2020 test function. We observed significant differences between the RCLSMAOA and other algorithms, except for CEC04. The main reason is that CEC04 is relatively simple compared to other functions, and most functions can find the optimal value.

To verify the ranking of the algorithm, we used Friedman detection. We ran each algorithm independently 30 times to take the average value, and the results are shown in Table 8 and Table 9. The dimension chosen for F23 functions here is 30. It can be noted that RCLSMAOA is still in the first position.

In this section, we conducted a more comprehensive data analysis of the algorithm’s performance using the Wilcoxon rank sum test and Friedman detection. We conclude that there are significant differences and good performance between the RCLSMAOA and most comparison algorithms for functions.

## 8. Engineering Issues

In this section, we will test the application of the RCLSMAOA in practical engineering problems in order to assess the quality and computational performance of RCLSMAOA in solving engineering problems and to explore whether it can achieve satisfactory results. This section will use five classic engineering problems to test the actual performance of the algorithm and compare it with other well-known optimization algorithms.

### 8.1. Pressure Vessel Design Problem

**Figure 7:**
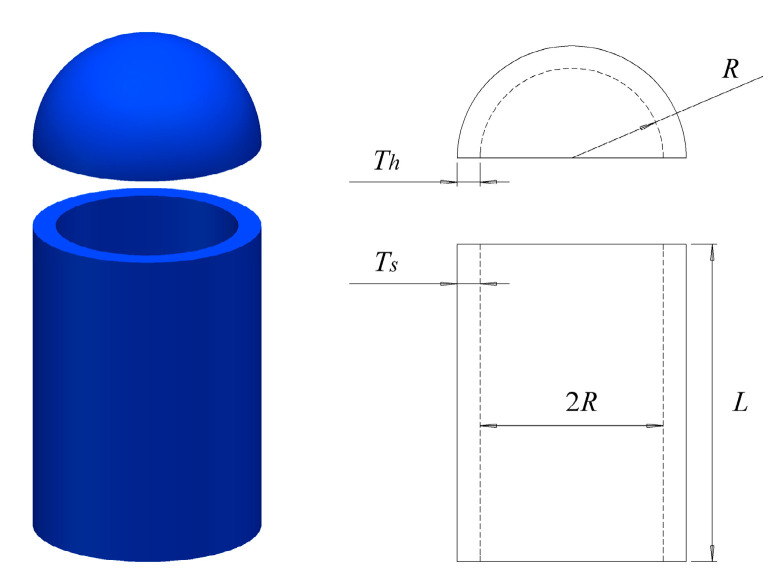
The pressure vessel design.

In practical survival problems, a common problem is pressure vessels. This issue aims to minimize the total cost of materials, forming, and welding for cylindrical containers. The structural schematic diagram is shown in Figure 7. This problem has four variables: shell thickness Ts, head thickness Th, internal radius R, and cylindrical section length L without considering the head.

The mathematical model of the pressure vessel design problem is as follows:

Consider:

(20) $$\vec{x}=\left[x_{1}\;\;\;x_{2}\;\;\;x_{3}\;\;\;x_{4}\right]=\left[T_{s}\;\;\;T_{h}\;\;\;R\;\;\;L\right]$$

Objective function:

(21) $$f\left(\vec{x}\right)=0.6224x_{1}x_{2}x_{3}+1.7781x_{2}x_{3}^{2}+3.1661x_{1}^{2}x_{4}+19.84x_{1}^{2}x_{3}$$

Subject to:

(22) $$g_{1}(\vec{x})=-x_{1}+0.0193x_{3}\leq 0$$

(23) $$g_{2}(\vec{x})=-x_{3}+0.00954x_{3}\leq 0$$

(24) $$g_{3}(\vec{x})=-\pi x_{3}^{2}x_{4}+\frac{2}{3}\pi x_{{}_{3}}^{3}+1296000\leq 0$$

(25) $$g_{4}(\vec{x})=-x_{4}-240\leq 0$$

Boundaries:

(26) $$\begin{array}{l} 0\leq x_{1}\leq 99,0\leq x_{2}\leq 99,10\leq x_{3}\leq 200\\ 10\leq x_{4}\leq 200 \end{array}$$

We can observe Table 10 to see the specific data of the RCLSMAOA on pressure vessel engineering issues. RCLSMAOA has Ts = 0.742433, Th = 0.370196, R = 40.31961, L = 200, COST = 5734.9131. Compared with other comparative algorithms, RCLSMAOA achieved the best results and achieved the optimal value of 200 on L. This means that RCLSMAOA can solve the engineering problem.

### 8.2. Speed Reducer Design Problem

**Figure 8:**
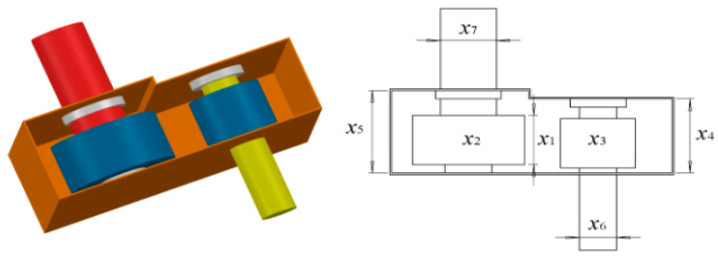
Model of speed reducer design.

The reducer is one of the key parts of the gearbox. In this study, we aim to achieve the minimum quality while meeting four design constraints and seven variables. The model structure is shown in Figure 8.

The mathematical model of reducer design is as follows:

Objective function:

(27) $$\begin{array}{l} f(\vec{x})=07854\times x_{1}\times x_{2}{}^{2}\times (3.3333\times x_{3}{}^{2}+14.9334\times x_{3}-\\ 43.0934)-1.508\times x_{1}\times (x_{6}{}^{2}+x_{7}{}^{2})+7.4777\times x_{6}{}^{3}+x_{7}{}^{3}+\\ 0.7854\times x_{4}\times x_{6}{}^{2}+x_{5}\times x_{7}{}^{2} \end{array}$$

Subject to:

(28) $$g_{1}(\vec{x})=\frac{27}{x_{1}\times x_{2}{}^{2}\times x_{3}}-1\leq 0$$

(29) $$g_{2}(\vec{x})=\frac{397.5}{x_{1}\times x_{2}{}^{2}\times x_{3}{}^{2}}-1\leq 0$$

(30) $$g_{3}(\vec{x})=\frac{1.93\times x_{4}{}^{3}}{x_{2}\times x_{3}\times x_{6}{}^{4}}-1\leq 0$$

(31) $$g_{4}(\vec{x})=\frac{1.93\times x_{5}{}^{3}}{x_{2}\times x_{3}\times x_{7}{}^{4}}-1\leq 0$$

(32) $$g_{5}(\vec{x})=\frac{1}{110\times x_{6}{}^{3}}\times \sqrt{{\left(\frac{745\times x_{4}}{x_{2}\times x_{3}}\right)}^{2}+16.9\times 10^{6}}-1\leq 0$$

(33) $$g_{6}(\vec{x})=\frac{1}{85\times x_{7}{}^{3}}\times \sqrt{{\left(\frac{745\times x_{5}}{x_{2}\times x_{3}}\right)}^{2}+16.9\times 10^{6}}-1\leq 0$$

(34) $$g_{7}(\vec{x})=\frac{x_{2}\times x_{3}}{40}-1\leq 0$$

(35) $$g_{8}(\vec{x})=\frac{5\times x_{2}}{x_{1}}-1\leq 0$$

(36) $$g_{9}(\vec{x})=\frac{x_{1}}{12\times x_{2}}-1\leq 0$$

(37) $$g_{10}(\vec{x})=\frac{1.5\times x_{6}+1.9}{x_{4}}-1\leq 0$$

(38) $$g_{11}(\vec{x})=\frac{1.1\times x_{7}+1.9}{x_{5}}-1\leq 0$$

Boundaries:

(39) $$\begin{array}{l} 2.6\leq x_{1}\leq 3.6,0.7\leq x_{2}\leq 0.8,17\leq x_{3}\leq 28,7.3\leq x_{4}\leq 8.3,\\ 7.3\leq x_{5}\leq 8.3,2.9\leq x_{6}\leq 3.9,5\leq x_{7}\leq 5.5 \end{array}$$

Table 11 shows that when x = [3.4975, 0.7, 17, 7.3, 7.8, 3.3500, 5.285], the minimum weight obtained by RCLSMAOA is 2995.437365, ranking first in the comparison algorithm. Observing experimental data, it can be seen that RCLSMAOA still performs well in relatively complex engineering problems.

### 8.3. Three-Bar Truss Design Problem

**Figure 9:**
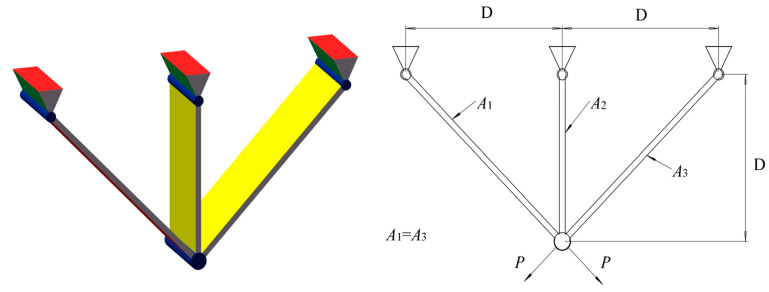
Pressure vessel design problem.

In the design problem of a three-bar truss, in order to minimize the weight constrained by stress, deflection, and buckling, it is necessary to operate on two-bar lengths to minimize volume while satisfying the three constraint conditions. It has two decision variables, namely the lengths A1 and A2 of the two rods, and its specific physical model is shown in Figure 9.

The mathematical formulation of this problem is shown below:

Consider:

(40) $$\vec{x}=[x_{1}\;x_{2}]=[A_{1}\;A_{2}]$$

Minimize:

(41) $$f(\vec{x})=(2\sqrt{2}x_{1}+x_{2})*l$$

Subject to:

(42) $$g_{1}(\vec{x})=\frac{\sqrt{2}x_{1}+x_{2}}{\sqrt{2}x_{1}^{2}+2x_{1}x_{2}}P-\sigma \leq 0,$$

(43) $$g_{2}(\vec{x})=\frac{x_{2}}{\sqrt{2}x_{1}^{2}+2x_{1}x_{2}}P-\sigma \leq 0,$$

(44) $$g_{3}(\vec{x})=\frac{1}{\sqrt{2}x_{1}+x_{1}}P-\sigma \leq 0,$$

(45) $$l=100\;\mathrm{cm},P=2{\;\mathrm{kN}/\mathrm{cm}}^{3},\sigma =2{\;\mathrm{kN}/\mathrm{cm}}^{3}$$

Variable Range:

(46) $$0\leq x_{1},x_{2}\leq 1,$$

The comparison results between RCLSMAOA and other algorithms in the design of three bar trusses are shown in Table 12. We observed that the data in the table are very close, indicating that it is difficult to optimize the problem better, but RCLSMAOA still achieved the best results.

### 8.4. Welded Beam Design Problem

**Figure 10:**
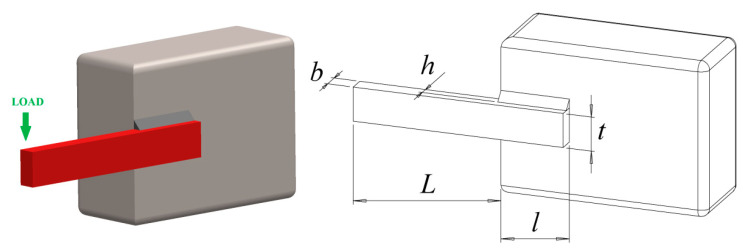
Model of welded beams design.

The welded beam design problem is a classic structural optimization problem and an important example in structural mechanics. This problem aims to minimize the steel plate’s total weight while satisfying four design variables of the connecting beam: thickness b, length L, height t, and width h. The detailed diagram of the welded beam is shown in Figure 10.

The mathematical model of welded beam design is as follows:

Consider:

(47) $$x=[x_{1}\;x_{2}\;x_{3}\;x_{4}]=[h\;l\;t\;b]$$

Objective function:

(48) $$f(x)=1.10471x_{1}^{2}x_{2}+0.04811x_{3}x_{4}(14.0+x_{2})$$

Subject to:

(49) $$g_{1}\left(\vec{x}\right)=\tau \left(\vec{x}\right)-\tau_{\max}\leq 0$$

(50) $$g_{2}\left(\vec{x}\right)=\sigma \left(\vec{x}\right)-\sigma_{\max}\leq 0$$

(51) $$g_{3}\left(\vec{x}\right)=\delta \left(\vec{x}\right)-\delta_{\max}\leq 0$$

(52) $$g_{4}\left(\vec{x}\right)=x_{1}-x_{4}\leq 0$$

(53) $$g_{5}\left(\vec{x}\right)=P-P_{c}\left(\vec{x}\right)\leq 0$$

(54) $$g_{6}\left(\vec{x}\right)=0.125-x_{1}\leq 0$$

(55) $$g_{7}\left(\vec{x}\right)=1.10471x_{1}^{2}+0.04811x_{3}x_{4}\left(14.0+x_{2}\right)-0.5\leq 0$$

where:

(56) $$\tau \left(\vec{x}\right)=\sqrt{{\left(\tau^{\prime }\right)}^{2}+2\tau^{\prime }\tau^{"}\frac{x_{2}}{2R}+\left(\tau^{"}\right)},\tau^{\prime }=\frac{P}{\sqrt{2x_{1}x_{2}}},\tau^{"}=\frac{MR}{J},$$

(57) $$M=P\left(L+\frac{x_{2}}{2}\right),R=\sqrt{\frac{x_{2}^{2}}{4}+{\left(\frac{x_{1}+x_{3}}{2}\right)}^{2}},\sigma \left(\vec{x}\right)=\frac{6PL}{x_{4}x_{3}^{2}},$$

(58) $$J=2\left\{\sqrt{2x_{1}x_{2}}\left[\frac{x_{x}^{2}}{4}+{\left(\frac{x_{1}+x_{3}}{2}\right)}^{2}\right]\right\},\delta \left(\vec{x}\right)=\frac{6PL^{3}}{Ex_{4}x_{3}^{2}},$$

(59) $$P_{c}\left(\vec{x}\right)=\frac{\sqrt[{4.013E}]{\frac{x_{3}^{2}x_{4}^{6}}{0}}}{L^{2}},\left(1-\frac{x_{3}}{2L}\sqrt{\frac{E}{4G}}\right),\left(1-\frac{x_{3}}{2L}\sqrt{\frac{E}{4G}}\right),$$

(60) P=6000lb,L=14  in,δmax=0.25 in,E=30×106   psi,

(61) $$\tau_{\max}=13600\;\;\;\;\;psi,\;and\;\;\;\;\;\sigma_{\max}=30000\;\;\;\;\;\;\;psi$$

Boundaries:

(62) $$0.1\leq x_{i}\leq 2,i=1,4;\;0.1\leq x_{i}\leq 10,i=2.3$$

The specific data are shown in Table 13. The same RCLSMAOA still performs well in engineering problems, and the weight of the welded beam also reaches the minimum value. The results indicate that the IBWO algorithm is reliable for solving the problem of welded beams.

### 8.5. Car Crashworthiness Design Problem

**Figure 11:**
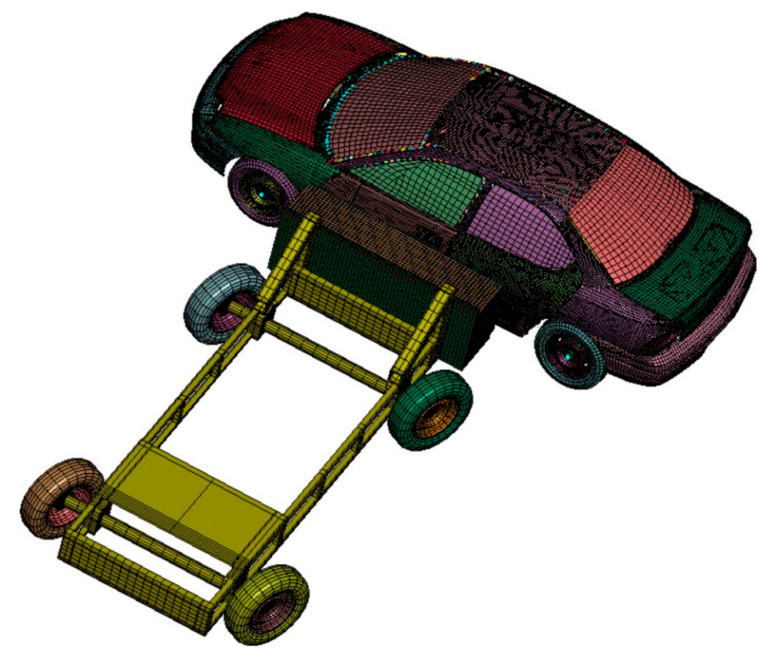
Car crashworthiness design.

The engineering issue refers to the safety performance of vehicles in a collision. This issue involves many aspects, including body structure, interior devices, and airbag systems. The car crashworthiness design problem is a very important issue in automotive design, which is directly related to the safety of passengers. The specific image is shown in Figure 11.

The mathematical formulation of this problem is shown below:

Minimize:

(63) $$f(\vec{x})=\mathrm{Weight},$$

Subject to:

(64) $$g_{1}(\vec{x})=F_{a}(\mathrm{load in abdomen})\leq 1\mathrm{kN},$$

(65) $$g_{2}(\vec{x})=V\times Cu_{}^{}(\mathrm{dummy upper chest})\leq 0.32_{}m/s,$$

(66) $$g_{3}(\vec{x})=V\times Cm_{}^{}(\mathrm{dummy middle chest})\leq 0.32_{}m/s,$$

(67) $$g_{4}(\vec{x})=V\times Cl_{}^{}(\mathrm{dummy lower chest})\leq 0.32_{}m/s,$$

(68) $$g_{5}(\vec{x})=\Delta_{\mathrm{ur}}{}_{}^{}(\mathrm{upper rib deflection})\leq 32_{}\mathrm{mm},$$

(69) $$g_{6}(\vec{x})=\Delta_{\mathrm{mr}}{}_{}^{}(\mathrm{middle rib deflection})\leq 32_{}\mathrm{mm},$$

(70) $$g_{7}(\vec{x})=\Delta_{\mathrm{lr}}{}_{}^{}(\mathrm{lower rib deflection})\leq 32_{}\mathrm{mm},$$

(71) $$g_{8}(\vec{x})=F_{}{\left({\mathrm{Public}}_{}^{}\mathrm{force}\right)}_{p}\leq 4_{}^{}\mathrm{kN},$$

(72) $$\begin{array}{l} g_{9}(\vec{x})=V_{\mathrm{MBP}}(\mathrm{Velocity of}\;V-\\ \mathrm{Pillar at middle point})\leq 9.9_{}\mathrm{mm}/\mathrm{ms}, \end{array}$$

(73) $$\begin{array}{l} g_{10}(\vec{x})=V_{\mathrm{FD}}(\mathrm{Velocity of front door at}\;V-\\ \mathrm{Pillar})\leq 15.7_{}\mathrm{mm}/\mathrm{ms}, \end{array}$$

Variable Range:

(74) $$0.5\leq x_{1}-x_{7}\leq 1.5,x_{8},x_{9}\in (0.192,0.345),-30\leq x_{10},x_{11}\leq 30,$$

The results of this engineering problem are shown in Table 14. In RCLSMAOA, X1, X3, X5, and X7 were all taken to the minimum value of 0.5, and the final weight was also the optimal value. This engineering problem shows that the RCLSMAOA still performs well in engineering problems with multiple variables and constraints.

## 9. Conclusions

This article fully considers the advantages and disadvantages of SMA and AOA optimization algorithms. It proposes a hybrid algorithm of slime mold and arithmetic optimization algorithm based on random center learning and restart mutation (RCLSMAOA). RCLSMAOA integrates the global search strategies of two algorithms. On this basis, a random center solution strategy is added to enhance the randomness of the algorithm, the effectiveness of global search, and the diversity of the algorithm population. The mutation strategy can enhance the convergence ability of the algorithm and further avoid the stagnation of the algorithm. Species reintroduction restart strategy can effectively avoid local optimization. The collaborative use of these strategies can help the RCLSMAOA enhance its optimization ability and maintain a good relationship between exploration and exploitation. In addition, we used the Wilcoxon rank sum test to test the significant differences between algorithms and achieved good results. Finally, five engineering experiments were conducted, and the RCLSMAOA provided an excellent solution.

From the experimental performance, convergence curve, and engineering problems, we can conclude that:

The RCLSMAOA proposed in this article combines the advantages of the SMA and AOA and effectively avoids the shortcomings of the two algorithms.

The newly proposed random center solution strategy can effectively address the shortcomings of RCLSMAOA and significantly enhance the algorithm’s global search ability.

The restart mutation strategy can improve the algorithm’s ability to overcome local optima and enhance the balance between exploration and exploitation.

By verifying the results of different test functions, the actual performance of the RCLSMAOA was effectively tested

Finally, by verifying five engineering problems, it can be concluded that the RCLSMAOA has good engineering application prospects.

This paper only studies the fusion of two optimization algorithms and adds three effective strategies. RCMSMAOA is still prone to local optimality in high dimensional space, and the convergence accuracy is not enough, and in some engineering problems did not show obvious advantages. In the future, we will further improve the performance of RCLSMAOA in practical engineering problems and improve the applicability of this algorithm in high-dimensional space. In future work, we will study the binary version of RCLSMAOA and use it to solve the feature selection problem.

## Figures and Tables

**Table 1:** Parameter settings for the comparative algorithms.

Algorithm	Parameter Settings
RCLSMAOA	*z* = 0.03; µ = 0.499; α = 5
AOA [11]	α = 5; *MOP_Max =* 1; *MOP_Min =* 0.2; µ = 0.499
SMA [15]	*z* = 0.03
ROA [45]	*C =* 0.1
SCA [46]	*a = 2*
WOA [47]	$\vec{A}=1\;;\;\vec{c}\in [-1,1]\;;\;b=0.75\;;\;l\in [-1,1]$
WMFO [42]	aϵ[1,2]; b = 1
AMVO-SCA [43]	W_max_ = 1; W_min_ = 0.2

**Table 2:** Results of benchmark functions (F1–F13) under 30 dimensions.

Fn	Metric	RCLSMAOA	AOA	SMA	ROA	SCA	WOA	WMFO	AMVO-SCA
F1	Best	0	1.77 × 10^−163^	0	0	2.34 × 10^−2^	2.96 × 10^−82^	3.31 × 10^−73^	5.59 × 10^−1^
	Mean	0	3.59 × 10^−22^	5.24 × 10^−306^	4.33 × 10^−306^	7.04	6.68 × 10^−72^	1.49 × 10^−54^	2.15
	Stg	0	1.97 × 10^−21^	0	0	1.15 × 10^1^	3.66 × 10^−71^	5.61 × 10^−54^	8.68 × 10^−1^
F2	Best	0	0	2.59 × 10^−273^	2.54 × 10^−18^3	4.56 × 10^−4^	4.88 × 10^−58^	3.86 × 10^−36^	2.56 × 10^−1^
	Mean	0	0	4.09 × 10^−157^	1.66 × 10^−162^	2.61 × 10^−2^	2.50 × 10^−51^	1.31 × 10^−26^	6.06 × 10^−1^
	Stg	0	0	2.24 × 10^−156^	6.67 × 10^−162^	2.68 × 10^−2^	6.92 × 10^−51^	5.00 × 10^−26^	1.96 × 10^−1^
F3	Best	0	4.73 × 10^−117^	0	0	1.50 × 10^3^	1.03 × 10^4^	7.53 × 10^−46^	4.74 × 10^1^
	Mean	0	5.09 × 10^−3^	3.79 × 10^−275^	7.70 × 10^−280^	7.07 × 10^3^	4.41 × 10^4^	3.58 × 10^1^	1.18 × 10^2^
	Stg	0	9.36 × 10^−3^	0	0	4.09 × 10^3^	1.49 × 10^4^	1.90 × 10^2^	4.05 × 10^1^
F4	Best	0	1.07 × 10^−54^	3.97 × 10^−283^	1.82 × 10^−176^	2.36 × 10^1^	1.91	2.11 × 10^−30^	5.16
	Mean	0	3.23 × 10^−2^	5.55 × 10^−138^	2.33 × 10^−159^	3.75 × 10^1^	4.96 × 10^1^	1.17 × 10^−10^	8.09
	Stg	0	1.86 × 10^−2^	3.04 × 10^−137^	1.28 × 10^−158^	7.62	2.73 × 10^1^	6.20 × 10^−10^	1.97
F5	Best	6.30 × 10^−5^	2.74 × 10^1^	4.46 × 10^−4^	2.61 × 10^1^	1.12 × 10^2^	2.70 × 10^1^	0	6.04 × 10^1^
	Mean	1.85 × 10^−2^	2.83 × 10^1^	5.16	2.70 × 10^1^	2.84 × 10^4^	2.80 × 10^1^	1.21 × 10^1^	1.37 × 10^2^
	Stg	2.27 × 10^−2^	3.45 × 10^−1^	9.59	5.69 × 10^−1^	5.48 × 10^4^	4.53 × 10^−1^	1.40 × 10^1^	1.12 × 10^2^
F6	Best	2.61 × 10^−7^	2.73	1.35 × 10^−5^	1.37 × 10^−2^	4.98	9.36 × 10^−2^	0	4.30
	Mean	3.59 × 10^−6^	3.17	5.77 × 10^−3^	1.17 × 10^−1^	2.35 × 10^1^	4.39 × 10^−1^	0	7.05
	Stg	2.99 × 10^−6^	2.28 × 10^−1^	3.57 × 10^−3^	1.42 × 10^−1^	2.99 × 10^1^	2.17 × 10^−1^	0	2.60
F7	Best	5.61 × 10^−7^	3.49 × 10^−6^	1.57 × 10^−5^	6.78 × 10^−6^	2.08 × 10^−2^	1.57 × 10^−4^	2.42 × 10^−6^	4.01 × 10^−2^
	Mean	4.30 × 10^−5^	6.04 × 10^−5^	1.84 × 10^−4^	1.60 × 10^−4^	1.55 × 10^−1^	4.62 × 10^−3^	2.96 × 10^−4^	6.01 × 10^−2^
	Stg	4.68 × 10^−5^	5.87 × 10^−5^	1.95 × 10^−4^	1.91 × 10^−4^	2.07 × 10^−1^	9.69 × 10^−3^	2.31 × 10^−4^	1.78 × 10^−2^
F8	Best	−1.26 × 10^4^	−6.32 × 10^3^	−1.26 × 10^4^	−1.26 × 10^4^	−4.24 × 10^3^	−1.26 × 10^4^	−2.37 × 10^+22^	−7.24 × 10^3^
	Mean	−1.26 × 10^4^	−5.21 × 10^3^	−1.26 × 10^4^	−1.24 × 10^4^	−3.69 × 10^3^	−1.05 × 10^4^	−1.42 × 10^+23^	−6.49 × 10^3^
	Stg	1.22	4.71 × 10^2^	4.26 × 10^−1^	4.31 × 10^2^	2.97 × 10^2^	1.76 × 10^3^	7.55 × 10^+23^	7.77 × 10^2^
F9	Best	0	0	0	0	2.84 × 10^−1^	0	0	6.28 × 10^1^
	Mean	0	0	0	0	4.16 × 10^1^	1.89 × 10^−15^	2.65 × 10^1^	9.28 × 10^1^
	Stg	0	0	0	0	3.30 × 10^1^	1.04 × 10^−14^	3.10 × 10^1^	2.26 × 10^1^
F10	Best	8.88 × 10^−16^	8.88 × 10^−16^	8.88 × 10^−16^	8.88 × 10^−16^	1.04 × 10^−1^	8.88 × 10^−16^	8.88 × 10^−16^	4.46
	Mean	8.88 × 10^−16^	8.88 × 10^−16^	8.88 × 10^−16^	8.88 × 10^−16^	1.53 × 10^1^	3.97 × 10^−15^	1.13 × 10^−15^	6.10
	Stg	0	0	0	0	7.95	2.42 × 10^−15^	1.30 × 10^−15^	8.36 × 10^−1^
F11	Best	0	1.39 × 10^−2^	0	0	3.92 × 10^−2^	0	0	8.05 × 10^−1^
	Mean	0	1.78 × 10^−1^	0	0	9.90 × 10^−1^	1.71 × 10^−2^	0	9.68 × 10^−1^
	Stg	0	1.31 × 10^−1^	0	0	3.80 × 10^−1^	6.81 × 10^−2^	0	6.45 × 10^−2^
F12	Best	5.17 × 10^−9^	4.44 × 10^−1^	2.83 × 10^−5^	2.39 × 10^−3^	2.34	3.37 × 10^−3^	1.57 × 10^−32^	7.35
	Mean	8.41 × 10^−8^	5.18 × 10^−1^	5.35 × 10^−3^	9.19 × 10^−3^	6.29 × 10^4^	1.97 × 10^−2^	1.04 × 10^−1^	1.11 × 10^1^
	Stg	8.95 × 10^−8^	4.96 × 10^−2^	6.30 × 10^−3^	4.46 × 10^−3^	2.73 × 10^5^	1.41 × 10^−2^	5.68 × 10^−1^	3.00
F13	Best	6.96 × 10^−8^	2.62	9.35 × 10^−6^	6.01 × 10^−3^	2.77	2.03 × 10^−1^	1.35 × 10^−32^	1.61 × 10^1^
	Mean	7.57 × 10^−7^	2.85	4.01 × 10^−3^	2.04 × 10^−1^	1.36 × 10^5^	5.37 × 10^−1^	1.80 × 10^−27^	2.91 × 10^1^
	Stg	9.70 × 10^−7^	8.55 × 10^−2^	3.20 × 10^−3^	1.33 × 10^−1^	3.76 × 10^5^	2.60 × 10^−1^	9.89 × 10^−27^	9.91

**Table 3:** Results of benchmark functions (F1–F13) under 500 dimensions.

Fn	Metric	RCLSMAOA	AOA	SMA	ROA	SCA	WOA	WMFO	AMVO-SCA
F1	Best	0	5.96 × 10^−1^	0	0	2.06 × 10^5^	1.70 × 10^−76^	2.80 × 10^−68^	7.37 × 10^−1^
	Mean	0	6.43 × 10^−1^	3.54 × 10^−259^	0	2.98 × 10^5^	1.75 × 10^−69^	2.15 × 10^−52^	2.32
	Stg	0	5.98 × 10^−2^	0	0	8.38 × 10^4^	3.89 × 10^−69^	1.15 × 10^−51^	1
F2	Best	0	2.47 × 10^−4^	9.02 × 10^−16^	1.50 × 10^−174^	9.36 × 10^1^	3.38 × 10^−51^	7.99 × 10^−37^	3.51 × 10^−1^
	Mean	0	2.74 × 10^−3^	6.76 × 10^−1^	3.35 × 10^−159^	1.84 × 10^2^	6.39 × 10^−48^	7.25 × 10^−22^	6.00 × 10^−1^
	Stg	0	2.28 × 10^−3^	9.50 × 10^−1^	7.50 × 10^−159^	6.74 × 10^1^	1.08 × 10^−47^	3.97 × 10^−21^	1.34 × 10^−1^
F3	Best	0	2.91 × 10^1^	0	2.49 × 10^−291^	6.53 × 10^6^	2.78 × 10^7^	3.05 × 10^−50^	3.78 × 10^1^
	Mean	0	5.28 × 10^1^	4.30 × 10^−208^	2.89 × 10^−279^	8.07 × 10^6^	3.90 × 10^7^	9.40	1.19 × 10^2^
	Stg	0	3.30 × 10^1^	0	0	1.73 × 10^6^	8.30 × 10^6^	4.41 × 10^1^	6.19 × 10^1^
F4	Best	0	1.76 × 10^−1^	1.20 × 10^−159^	4.65 × 10^−170^	9.88 × 10^1^	5.20 × 10^1^	8.09 × 10^−32^	5.36
	Mean	0	2.00 × 10^−1^	2.89 × 10^−120^	2.18 × 10^−15^6	9.92 × 10^1^	7.28 × 10^1^	1.03 × 10^−10^	8.33
	Stg	0	4.69 × 10^−2^	6.25 × 10^−120^	4.85 × 10^−15^6	2.93 × 10^−1^	2.00 × 10^1^	5.64 × 10^−10^	2.02
F5	Best	1.37 × 10^−5^	4.99 × 10^2^	3.27 × 10^1^	4.94 × 10^2^	2.03 × 10^9^	4.96 × 10^2^	0	7.12 × 10^1^
	Mean	7.52 × 10^−2^	4.99 × 10^2^	3.70 × 10^2^	4.95 × 10^2^	2.32 × 10^9^	4.97 × 10^2^	8.35	1.35 × 10^2^
	Stg	8.06 × 10^−2^	9.93 × 10^−2^	2.03 × 10^2^	2.87 × 10^−1^	3.50 × 10^8^	4.41 × 10^−1^	1.30 × 10^1^	7.25 × 10^1^
F6	Best	1.51 × 10^−6^	1.13 × 10^2^	8.25 × 10^−1^	1.38 × 10^1^	1.30 × 10^5^	2.53 × 10^1^	0	4.06
	Mean	7.01 × 10^−3^	1.16 × 10^2^	5.24 × 10^1^	1.98 × 10^1^	2.25 × 10^5^	3.79 × 10^1^	0	6.94
	Stg	9.92 × 10^−3^	1.80	4.74 × 10^1^	5.45	8.80 × 10^4^	1.21 × 10^1^	0	2.07
F7	Best	2.45 × 10^−7^	8.86 × 10^−5^	8.56 × 10^−5^	1.25 × 10^−4^	1.60 × 10^4^	1.66 × 10^−3^	3.12 × 10^−5^	3.40 × 10^−2^
	Mean	2.63 × 10^−5^	1.37 × 10^−4^	7.06 × 10^−4^	3.96 × 10^−4^	1.79 × 10^4^	1.21 × 10^−2^	2.90 × 10^−4^	6.14 × 10^−2^
	Stg	2.30 × 10^−5^	4.50 × 10^−5^	8.20 × 10^−4^	2.59 × 10^−4^	2.22 × 10^3^	1.66 × 10^−2^	2.09 × 10^−4^	1.69 × 10^−2^
F8	Best	−2.09 × 10^5^	−2.37 × 10^4^	−2.09 × 10^5^	−2.09 × 10^5^	−1.58 × 10^4^	−2.06 × 10^5^	−8.54 × 10^24^	−7.91 × 10^3^
	Mean	−2.09 × 10^5^	−2.18 × 10^4^	−2.09 × 10^5^	−1.99 × 10^5^	−1.47 × 10^4^	−1.76 × 10^5^	−2.85 × 10^23^	−6.41 × 10^3^
	Stg	1.77 × 10^−1^	2.03 × 10^3^	2.34 × 10^2^	1.55 × 10^4^	6.84 × 10^2^	4.11 × 10^4^	1.56 × 10^24^	6.24 × 10^2^
F9	Best	0	0	0	0	5.17 × 10^2^	0	0	5.17 × 10^1^
	Mean	0	6.93 × 10^−6^	0	0	1.42 × 10^3^	6.06 × 10^−14^	1.19 × 10^1^	9.26 × 10^1^
	Stg	0	6.72 × 10^−6^	0	0	5.55 × 10^2^	3.32 × 10^−13^	2.43 × 10^1^	2.28 × 10^1^
F10	Best	8.88 × 10^−16^	7.44 × 10^−3^	8.88 × 10^−16^	8.88 × 10^−16^	8.07	8.88 × 10^−16^	8.88 × 10^−16^	5.15
	Mean	8.88 × 10^−16^	8.12 × 10^−3^	8.88 × 10^−16^	8.88 × 10^−16^	1.92 × 10^1^	4.32 × 10^−15^	8.88 × 10^−16^	6.14
	Stg	0	3.45 × 10^−4^	0	0	3.62	2.38 × 10^−15^	0	6.10 × 10^−1^
F11	Best	0	6.43 × 10^3^	0	0	9.67 × 10^2^	0	0	7.55 × 10^−1^
	Mean	0	1.06 × 10^4^	0	0	2.02 × 10^3^	3.70 × 10−18	0	9.87 × 10^−1^
	Stg	0	2.97 × 10^3^	0	0	7.53 × 10^2^	2.03 × 10−17	0	6.07 × 10^−2^
F12	Best	4.18 × 10^−13^	1.06	2.34 × 10^−5^	1.43 × 10^−2^	3.40 × 10^9^	3.93 × 10^−2^	1.57 × 10^−32^	7.08
	Mean	2.20 × 10^−7^	1.08	2.60 × 10^−2^	3.97 × 10^−2^	5.72 × 10^9^	1.06 × 10^−1^	1.57 × 10^−32^	1.02 × 10^1^
	Stg	2.92 × 10^−7^	1.36 × 10^−2^	9.59 × 10^−2^	2.25 × 10^−2^	1.47 × 10^9^	5.14 × 10^−2^	5.57 × 10^−48^	2.56
F13	Best	6.02 × 10^−11^	5.01 × 10^1^	3.61 × 10^−3^	3.37	5.32 × 10^9^	8.64	1.35 × 10^−32^	1.82 × 10^1^
	Mean	1.79 × 10^−3^	5.02 × 10^1^	2.87	9.03	1.03 × 10^10^	2.00 × 10^1^	1.35 × 10^−32^	3.00 × 10^1^
	Stg	3.77 × 10^−3^	4.33 × 10^−2^	8.97	2.72	2.39 × 10^9^	5.75	5.57 × 10^−48^	8.14

**Table 4:** Results of benchmark functions (F14–F23).

Fn	Metric	RCLSMAOA	AOA	SMA	ROA	SCA	WOA	WMFO	AMVO-SCA
F14	Best	9.98 × 10^−1^	9.98 × 10^−1^	9.98 × 10^−1^	9.98 × 10^−1^	9.98 × 10^−1^	9.98 × 10^−1^	9.98 × 10^−1^	9.98 × 10^−1^
	Mean	9.98 × 10^−1^	9.49	9.98 × 10^−1^	3.35	1.60	3.71	4.23	5.74
	Stg	0	3.63	8.61 × 10^−13^	4.01	9.23 × 10^−1^	4.02	3.92	4.34
F15	Best	3.07 × 10^−4^	3.77 × 10^−4^	3.08 × 10^−4^	3.08 × 10^−4^	4.92 × 10^−4^	3.58 × 10^−4^	3.07 × 10^−4^	3.68 × 10^−4^
	Mean	3.45 × 10^−4^	1.69 × 10^−2^	6.23 × 10^−4^	5.04 × 10^−4^	1.10 × 10^−3^	6.97 × 10^−4^	4.37 × 10^−4^	1.38 × 10^−3^
	Stg	9.47 × 10^−5^	3.25 × 10^−2^	3.04 × 10^−4^	3.18 × 10^−4^	3.56 × 10^−4^	4.54 × 10^−4^	2.96 × 10^−4^	1.10 × 10^−3^
F16	Best	−1.03	−1.03	−1.03	−1.03	−1.03	−1.03	−1.03	−1.03
	Mean	−1.03	−1.03	−1.03	−1.03	−1.03	−1.03	−1.03	−1.03
	Stg	6.78 × 10^−16^	1.34 × 10^−7^	1.59 × 10^−9^	5.27 × 10^−8^	5.73 × 10^−5^	2.87 × 10^−9^	5.80 × 10^−10^	5.27 × 10^−3^
F17	Best	3.98 × 10^−1^	3.98 × 10^−1^	3.98 × 10^−1^	3.98 × 10^−1^	3.98 × 10^−1^	3.98 × 10^−1^	3.98 × 10^−1^	3.98 × 10^−1^
	Mean	3.98 × 10^−1^	3.98 × 10^−1^	3.98 × 10^−1^	3.98 × 10^−1^	4.00 × 10^−1^	3.98 × 10^−1^	3.98 × 10^−1^	3.98 × 10^−1^
	Stg	0	1.36 × 10^−7^	2.84 × 10^−8^	1.32 × 10^−5^	1.55 × 10^−3^	5.72 × 10^−6^	1.02 × 10^−8^	7.57 × 10^−4^
F18	Best	3	3	3	3	3	3	3	3
	Mean	3	1.34 × 10^1^	3	3	3	3	3	3
	Stg	2.08 × 10^−15^	2.01 × 10^1^	7.57 × 10^−11^	6.18 × 10^−5^	8.12 × 10^−5^	1.05 × 10^−2^	5.99 × 10^−6^	6.46 × 10^−13^
F19	Best	−3.86	−3.86	−3.86	−3.86	−3.86	−3.86	−3.86	−3.86
	Mean	−3.86	−3.85	−3.86	−3.86	−3.85	−3.86	−3.86	−3.86
	Stg	2.71 × 10^−15^	5.82 × 10^−3^	1.90 × 10^−6^	2.77 × 10^−3^	6.12 × 10^−3^	1.07 × 10^−2^	3.39 × 10^−3^	1.36 × 10^−2^
F20	Best	−3.32	−3.16	−3.32	−3.32	−3.13	−3.32	−3.32	−3.32
	Mean	−3.29	−3.02	−3.24	−3.21	−2.87	−3.20	−3.13	−3.01
	Stg	5.35 × 10^−2^	9.55 × 10^−2^	5.58 × 10^−2^	1.42 × 10^−1^	3.47 × 10^−1^	1.73 × 10^−1^	3.13 × 10^−1^	3.59 × 10^−1^
F21	Best	−1.02 × 10^1^	−5.16	−1.02 × 10^1^	−1.02 × 10^1^	−5.90	−1.01 × 10^1^	−1.02 × 10^1^	−1.01 × 10^1^
	Mean	−1.02 × 10^1^	−3.62	−1.02 × 10^1^	−1.01 × 10^1^	−2.40	−7.60	−5.23	−4.72
	Stg	6.96 × 10^−15^	1.06	4.55 × 10^−4^	1.58 × 10^−2^	1.86	2.81	9.31 × 10^−1^	2.63
F22	Best	−1.04 × 10^1^	−7.58	−1.04 × 10^1^	−1.04 × 10^1^	−6.85	−1.04 × 10^1^	−1.04 × 10^1^	−1.04 × 10^1^
	Mean	−1.04 × 10^1^	−4.29	−1.04 × 10^1^	−1.04 × 10^1^	−3.69	−7.69	−6.26	−5.89
	Stg	1.19 × 10^−15^	1.23	2.55 × 10^−4^	1.59 × 10^−2^	1.86	3.21	2.71	3.10
F23	Best	−1.05 × 10^1^	−8.42	−1.05 × 10^1^	−1.05 × 10^1^	−8.38	−1.05 × 10^1^	−1.05 × 10^1^	−1.05 × 10^1^
	Mean	−1.05 × 10^1^	−4.06	−1.05 × 10^1^	−1.05 × 10^1^	−3.86	−7.34	−7.29	−5.23
	Stg	1.78 × 10^−15^	1.72	3.91 × 10^−4^	2.00 × 10^−2^	1.87	3.09	2.69	3.07

**Table 5:** Results of benchmark functions (F14–F23).

CEC	Metric	RCLSMAOA	AOA	SMA	ROA	SCA	WOA	WMFO	AMVO-SCA
CEC_01	mid	1.00 × 10^2^	2.99 × 10^9^	1.05 × 10^2^	1.05 × 10^9^	4.08 × 10^8^	5.00 × 10^6^	1.19 × 10^3^	3.15 × 10^3^
	mean	1.80 × 10^3^	1.02 × 10^10^	7.28 × 10^3^	5.69 × 10^9^	1.10 × 10^9^	7.74 × 10^7^	1.57 × 10^5^	8.64 × 10^8^
	std	1.88 × 10^3^	4.13 × 10^9^	5.00 × 10^3^	3.26 × 10^9^	5.80 × 10^8^	1.13 × 10^8^	4.30 × 10^5^	1.43 × 10^9^
CEC_02	mid	1.10 × 10^3^	1.83 × 10^3^	1.34 × 10^3^	1.77 × 10^3^	1.75 × 10^3^	1.63 × 10^3^	1.46 × 10^3^	1.57 × 10^3^
	mean	1.42 × 10^3^	2.22 × 10^3^	1.77 × 10^3^	2.49 × 10^3^	2.54 × 10^3^	2.24 × 10^3^	1.98 × 10^3^	2.00 × 10^3^
	std	1.33 × 10^2^	2.30 × 10^2^	2.52 × 10^2^	3.17 × 10^2^	2.73 × 10^2^	3.44 × 10^2^	3.62 × 10^2^	3.44 × 10^2^
CEC_03	mid	7.11 × 10^2^	7.70 × 10^2^	7.18 × 10^2^	7.71 × 10^2^	7.56 × 10^2^	7.52 × 10^2^	7.22 × 10^2^	7.30 × 10^2^
	mean	7.18 × 10^2^	7.96 × 10^2^	7.32 × 10^2^	8.17 × 10^2^	7.86 × 10^2^	7.97 × 10^2^	7.45 × 10^2^	7.65 × 10^2^
	std	2.75	1.56 × 10^1^	9.63	2.46 × 10^1^	1.41 × 10^1^	2.76 × 10^1^	1.59 × 10^1^	3.23 × 10^1^
CEC_04	mid	1.90 × 10^3^	1.90 × 10^3^	1.90 × 10^3^	1.90 × 10^3^	1.90 × 10^3^	1.90 × 10^3^	1.90 × 10^3^	1.90 × 10^3^
	mean	1.90 × 10^3^	1.90 × 10^3^	1.90 × 10^3^	1.90 × 10^3^	1.90 × 10^3^	1.90 × 10^3^	1.90 × 10^3^	1.90 × 10^3^
	std	0	0	0	0	1.09	2.56 × 10^−1^	5.83 × 10^−1^	2.58
CEC_05	mid	1.70 × 10^3^	9.15 × 10^3^	2.46 × 10^3^	4.58 × 10^3^	1.23 × 10^4^	7.71 × 10^3^	6.75 × 10^3^	3.67 × 10^3^
	mean	2.91 × 10^3^	4.49 × 10^5^	2.69 × 10^4^	4.77 × 10^5^	6.57 × 10^4^	2.59 × 10^5^	3.36 × 10^5^	3.36 × 10^5^
	std	1.62 × 10^3^	3.28 × 10^5^	6.82 × 10^4^	3.36 × 10^5^	6.78 × 10^4^	5.10 × 10^5^	5.16 × 10^5^	3.66 × 10^5^
CEC_06	mid	1.60 × 10^3^	1.76 × 10^3^	1.61 × 10^3^	1.65 × 10^3^	1.69 × 10^3^	1.65 × 10^3^	1.61 × 10^3^	1.60 × 10^3^
	mean	1.65 × 10^3^	2.15 × 10^3^	1.77 × 10^3^	1.96 × 10^3^	1.86 × 10^3^	1.89 × 10^3^	1.82 × 10^3^	1.86 × 10^3^
	std	5.85 × 10^1^	1.99 × 10^2^	1.05 × 10^2^	1.52 × 10^2^	9.03 × 10^1^	1.25 × 10^2^	1.39 × 10^2^	1.74 × 10^2^
CEC_07	mid	2.10 × 10^3^	4.05 × 10^3^	2.33 × 10^3^	2.98 × 10^3^	5.60 × 10^3^	8.70 × 10^3^	3.43 × 10^3^	2.76 × 10^3^
	mean	2.62 × 10^3^	1.04 × 10^6^	9.48 × 10^3^	3.66 × 10^5^	1.72 × 10^4^	7.75 × 10^5^	1.76 × 10^5^	5.75 × 10^5^
	std	7.73 × 10^2^	2.14 × 10^6^	9.22 × 10^3^	1.02 × 10^6^	1.06 × 10^4^	2.07 × 10^6^	3.79 × 10^5^	2.97 × 10^6^
CEC_08	mid	2.20 × 10^3^	2.59 × 10^3^	2.30 × 10^3^	2.38 × 10^3^	2.33 × 10^3^	2.31 × 10^3^	2.23 × 10^3^	2.30 × 10^3^
	mean	2.30 × 10^3^	3.07 × 10^3^	2.46 × 10^3^	2.71 × 10^3^	2.41 × 10^3^	2.38 × 10^3^	2.40 × 10^3^	2.50 × 10^3^
	std	1.99 × 10^1^	3.35 × 10^2^	3.69 × 10^2^	3.50 × 10^2^	4.66 × 10^1^	2.92 × 10^2^	3.80 × 10^2^	3.58 × 10^2^
CEC_09	mid	2.40 × 10^3^	2.66 × 10^3^	2.50 × 10^3^	2.60 × 10^3^	2.57 × 10^3^	2.57 × 10^3^	2.74 × 10^3^	2.50 × 10^3^
	mean	2.72 × 10^3^	2.88 × 10^3^	2.75 × 10^3^	2.81 × 10^3^	2.79 × 10^3^	2.78 × 10^3^	2.76 × 10^3^	2.76 × 10^3^
	std	6.67 × 10^1^	8.73 × 10^1^	3.82 × 10^1^	8.55 × 10^1^	4.37 × 10^1^	5.17 × 10^1^	2.41 × 10^1^	7.40 × 10^1^
CEC_10	mid	2.90 × 10^3^	2.99 × 10^3^	2.90 × 10^3^	2.97 × 10^3^	2.94 × 10^3^	2.91 × 10^3^	2.90 × 10^3^	2.91 × 10^3^
	mean	2.93 × 10^3^	3.38 × 10^3^	2.95 × 10^3^	3.25 × 10^3^	2.99 × 10^3^	2.98 × 10^3^	2.94 × 10^3^	2.97 × 10^3^
	std	2.17 × 10^1^	2.89 × 10^2^	3.18 × 10^1^	2.57 × 10^2^	3.12 × 10^1^	9.68 × 10^1^	2.93 × 10^1^	6.47 × 10^1^

**Table 6:** Experimental results of Wilcoxon rank sum test for F23 functions.

F23	dim	RCLSMAOA vs. AOA	RCLSMAOA vs. SMA	RCLSMAOA vs. ROA	RCLSMAOA vs. SCA	RCLSMAOA vs. WOA	RCLSMAOA vs. WMFO	RCLSMAOA vs. AMVO-SCA
F1	30	1.73 × 10^−6^	5.00 × 10^−1^	1	1.73 × 10^−6^	1.73 × 10^−6^	6.10 × 10^−5^	6.10 × 10^−5^
	500	1.73 × 10^−6^	1.22 × 10^−4^	5.00 × 10^−1^	1.73 × 10^−6^	1.73 × 10^−6^	6.10 × 10^−5^	6.10 × 10^−5^
F2	30	1	1.73 × 10^−6^	1.73 × 10^−6^	1.73 × 10^−6^	1.73 × 10^−6^	6.10 × 10^−5^	6.10 × 10^−5^
	500	1	1.73 × 10^−6^	1.73 × 10^−6^	1.73 × 10^−6^	1.73 × 10^−6^	6.10 × 10^−5^	6.10 × 10^−5^
F3	30	1.73 × 10^−6^	1	1.95 × 10^−3^	1.73 × 10^−6^	1.73 × 10^−6^	6.10 × 10^−5^	6.10 × 10^−5^
	500	1.73 × 10^−6^	1	6.10 × 10^−5^	1.73 × 10^−6^	1.73 × 10^−6^	6.10 × 10^−5^	6.10 × 10^−5^
F4	30	1.73 × 10^−6^	1.73 × 10^−6^	1.73 × 10^−6^	1.73 × 10^−6^	1.73 × 10^−6^	6.10 × 10^−5^	6.10 × 10^−5^
	500	1.73 × 10^−6^	1.73 × 10^−6^	1.73 × 10^−6^	1.73 × 10^−6^	1.73 × 10^−6^	6.10 × 10^−5^	6.10 × 10^−5^
F5	30	1.73 × 10^−6^	1.92 × 10^−6^	1.73 × 10^−6^	1.73 × 10^−6^	1.73 × 10^−6^	4.21 × 10^−1^	6.10 × 10^−5^
	500	1.73 × 10^−6^	2.88 × 10^−6^	1.73 × 10^−6^	1.73 × 10^−6^	1.73 × 10^−6^	8.04 × 10^−1^	6.10 × 10^−5^
F6	30	1.73 × 10^−6^	1.73 × 10^−6^	1.73 × 10^−6^	1.73 × 10^−6^	1.73 × 10^−6^	6.10 × 10^−5^	6.10 × 10^−5^
	500	1.73 × 10^−6^	1.73 × 10^−6^	1.73 × 10^−6^	1.73 × 10^−6^	1.73 × 10^−6^	6.10 × 10^−5^	6.10 × 10^−5^
F7	30	8.61 × 10^−1^	2.96 × 10^−3^	1.11 × 10^−2^	1.73 × 10^−6^	1.73 × 10^−6^	1.22 × 10^−4^	6.10 × 10^−5^
	500	2.99 × 10^−1^	4.53 × 10^−4^	3.61 × 10^−3^	1.73 × 10^−6^	2.60 × 10^−6^	6.10 × 10^−4^	6.10 × 10^−5^
F8	30	1.73 × 10^−6^	3.16 × 10^−2^	8.13 × 10^−1^	1.73 × 10^−6^	1.92 × 10^−6^	6.10 × 10^−5^	6.10 × 10^−5^
	500	1.73 × 10^−6^	1.04 × 10^−2^	4.45 × 10^−5^	1.73 × 10^−6^	2.35 × 10^−6^	6.10 × 10^−5^	6.10 × 10^−5^
F9	30	1	1	1	1.73 × 10^−6^	1	3.13 × 10^−2^	6.10 × 10^−5^
	500	1	1	1	1.73 × 10^−6^	2.50 × 10^−1^	7.81 × 10^−3^	6.10 × 10^−5^
F10	30	1	1	1	1.73 × 10^−6^	9.90 × 10^−6^	1	6.10 × 10^−5^
	500	1.73 × 10^−6^	1	1	1.73 × 10^−6^	5.00 × 10^−1^	1	6.10 × 10^−5^
F11	30	1.73 × 10^−6^	1	1	1.73 × 10^−6^	1	1	6.10 × 10^−5^
	500	1.73 × 10^−6^	1.73 × 10^−6^	1.73 × 10^−6^	1.73 × 10^−6^	1.73 × 10^−6^	1	6.10 × 10^−5^
F12	30	1.73 × 10^−6^	1.73 × 10^−6^	1.73 × 10^−6^	1.73 × 10^−6^	1.73 × 10^−6^	6.10 × 10^−5^	6.10 × 10^−5^
	500	1.73 × 10^−6^	1.73 × 10^−6^	1.73 × 10^−6^	1.73 × 10^−6^	1.73 × 10^−6^	6.10 × 10^−5^	6.10 × 10^−5^
F13	30	1.73 × 10^−6^	1.73 × 10^−6^	1.73 × 10^−6^	1.73 × 10^−6^	1.73 × 10^−6^	6.10 × 10^−5^	6.10 × 10^−5^
	500	1.73 × 10^−6^	1.73 × 10^−6^	1.73 × 10^−6^	1.73 × 10^−6^	1.73 × 10^−6^	6.10 × 10^−5^	6.10 × 10^−5^
F14	2	1.73 × 10^−6^	1.73 × 10^−6^	1.73 × 10^−6^	1.73 × 10^−6^	1.73 × 10^−6^	5.00 × 10^−1^	6.10 × 10^−5^
F15	4	1.92 × 10^−6^	4.45 × 10^−5^	9.32 × 10^−6^	1.73 × 10^−6^	2.35 × 10^−6^	6.10 × 10^−5^	1.07 × 10^−1^
F16	2	1.73 × 10^−6^	1.73 × 10^−6^	1.73 × 10^−6^	1.73 × 10^−6^	1.73 × 10^−6^	1	6.10 × 10^−5^
F17	2	1.73 × 10^−6^	1.73 × 10^−6^	1.73 × 10^−6^	1.73 × 10^−6^	1.73 × 10^−6^	1	6.10 × 10^−5^
F18	5	1.73 × 10^−6^	1.73 × 10^−6^	1.73 × 10^−6^	1.73 × 10^−6^	1.73 × 10^−6^	9.77 × 10^−4^	6.10 × 10^−5^
F19	3	1.73 × 10^−6^	1.73 × 10^−6^	1.73 × 10^−6^	1.73 × 10^−6^	1.73 × 10^−6^	1	6.10 × 10^−5^
F20	6	1.73 × 10^−6^	6.32 × 10^−5^	3.52 × 10^−6^	1.73 × 10^−6^	4.53 × 10^−4^	4.03 × 10^−3^	4.21 × 10^−1^
F21	4	1.73 × 10^−6^	1.73 × 10^−6^	1.73 × 10^−6^	1.73 × 10^−6^	1.73 × 10^−6^	3.13 × 10^−2^	6.10 × 10^−5^
F22	4	1.73 × 10^−6^	1.73 × 10^−6^	1.73 × 10^−6^	1.73 × 10^−6^	1.73 × 10^−6^	7.81 × 10^−3^	6.10 × 10^−5^
F23	4	1.73 × 10^−6^	1.73 × 10^−6^	1.73 × 10^−6^	1.73 × 10^−6^	1.73 × 10^−6^	3.13 × 10^−2^	6.10 × 10^−5^

**Table 7:** Experimental results of Wilcoxon rank sum test for CEC2020 functions.

F23	dim	RCLSMAOA vs. AOA	RCLSMAOA vs. SMA	RCLSMAOA vs. ROA	RCLSMAOA vs. SCA	RCLSMAOA vs. WOA	RCLSMAOA vs. WMFO	RCLSMAOA vs. AMVO-SCA
CEC01	10	1.73 × 10^−6^	6.34 × 10^−6^	1.73 × 10^−6^	1.73 × 10^−6^	1.73 × 10^−6^	4.27 × 10^−3^	6.10 × 10^−5^
CEC02	10	1.73 × 10^−6^	1.92 × 10^−6^	1.73 × 10^−6^	1.73 × 10^−6^	1.73 × 10^−6^	3.36 × 10^−3^	3.05 × 10^−4^
CEC03	10	1.73 × 10^−6^	1.73 × 10^−6^	1.73 × 10^−6^	1.73 × 10^−6^	1.73 × 10^−6^	1.22 × 10^−4^	6.10 × 10^−5^
CEC04	10	1.73 × 10^−6^	1	1	1.73 × 10^−6^	1	3.13 × 10^−2^	6.10 × 10^−5^
CEC05	10	1.73 × 10^−6^	1.73 × 10^−6^	1.73 × 10^−6^	1.73 × 10^−6^	1.73 × 10^−6^	1.22 × 10^−4^	6.10 × 10^−5^
CEC06	10	1.73 × 10^−6^	1.73 × 10^−6^	1.73 × 10^−6^	1.73 × 10^−6^	1.73 × 10^−6^	4.27 × 10^−4^	6.10 × 10^−5^
CEC07	10	1.73 × 10^−6^	1.92 × 10^−6^	1.73 × 10^−6^	1.73 × 10^−6^	1.73 × 10^−6^	1.16 × 10^−3^	2.62 × 10^−3^
CEC08	10	1.73 × 10^−6^	2.60 × 10^−6^	1.73 × 10^−6^	1.73 × 10^−6^	1.73 × 10^−6^	6.10 × 10^−5^	6.10 × 10^−5^
CEC09	10	1.73 × 10^−6^	1.73 × 10^−6^	1.73 × 10^−6^	1.73 × 10^−6^	1.73 × 10^−6^	8.33 × 10^−2^	8.33 × 10^−2^
CEC10	10	1.73 × 10^−6^	1.13 × 10^−5^	1.73 × 10^−6^	1.73 × 10^−6^	1.73 × 10^−6^	2.56 × 10^−2^	3.53 × 10^−2^

**Table 8:** Friedman ranked F23 functions.

F	RCLSMAOA	AOA	SMA	ROA	SCA	WOA	WMFO	AMVO-SCA
F1	1.933333333	4.266666667	1.983333333	2.083333333	7.666666667	5.866666667	4.866666667	7.333333333
F2	1.5	1.5	3.2	3.8	7	5	6	8
F3	1.5	4.3	1.5	3	7	8	4.7	6
F4	1	4.9	2.166666667	2.833333333	7	8	4.1	6
F5	1.7	5.1	2.866666667	3.666666667	7.966666667	5.9	1.766666667	7.033333333
F6	2	5.566666667	3	4	7.633333333	5.533333333	1	7.266666667
F7	2.333333333	1.8	3.866666667	2.966666667	7.533333333	6.3	4.1	7.1
F8	3	6.8	3	3	8	5.4	1	5.8
F9	3.133333333	3.133333333	3.133333333	3.133333333	6.866666667	3.683333333	5.05	7.866666667
F10	3.116666667	3.116666667	3.116666667	3.116666667	7.666666667	5.416666667	3.116666667	7.333333333
F11	2.85	5.866666667	2.85	2.85	7.3	3.9	2.85	7.533333333
F12	2	5.733333333	3.1	3.9	7.466666667	5.266666667	1	7.533333333
F13	2	6	3	4	7.8	5	1	7.2
F14	1.416666667	6.933333333	3	4.833333333	4.866666667	6.733333333	1.75	6.466666667
F15	1.566666667	6.166666667	4.1	2.7	5.566666667	5.666666667	6.266666667	3.966666667
F16	1.166666667	6.6	3.8	5.733333333	7.966666667	4.866666667	1.833333333	4.033333333
F17	1.5	4.766666667	3.6	5.866666667	7.5	7.5	1.5	3.766666667
F18	1.033333333	5.1	3.066666667	6.2	6.666666667	6.866666667	1.966666667	5.1
F19	1.3	6.5	3.033333333	5	6.3	7.333333333	1.7	4.833333333
F20	1.3	6.4	3.833333333	4.166666667	7.233333333	7.266666667	2.633333333	3.166666667
F21	1.033333333	6.266666667	2.733333333	3.833333333	7.466666667	5.933333333	4	4.733333333
F22	1.116666667	6.9	3.4	4.266666667	6.9	6.7	2.783333333	3.933333333
F23	1.083333333	6.8	3.366666667	4.266666667	6.8	6.533333333	2.583333333	4.566666667
Avg Rank	1.7644	5.5298	3.0746	3.8789	7.1376	6.0289	2.9376	5.9376
Final Rank	1	5	3	4	8	7	2	6

**Table 9:** Friedman ranked CEC2020 functions.

CEC2020	RCLSMAOA	AOA	SMA	ROA	SCA	WOA	WMFO	AMVO-SCA
CEC2020_01	1.466666667	7.6	2.133333333	5.166666667	5.266666667	7.366666667	2.666666667	4.333333333
CEC2020_02	1.233333333	5.166666667	3.066666667	4.766666667	6.933333333	7.566666667	3.633333333	3.633333333
CEC2020_03	1.066666667	7	2.4	4.766666667	5.766666667	7.633333333	2.966666667	4.4
CEC2020_04	3.383333333	3.383333333	3.383333333	3.383333333	5.683333333	3.766666667	5.083333333	7.933333333
CEC2020_05	1.133333333	7.066666667	3.3	4.533333333	4.466666667	5.4	5.6	4.5
CEC2020_06	1.4	6.8	2.7	4.6	3.833333333	7.1	4.466666667	5.1
CEC2020_07	1.8	6.7	3.6	4.133333333	4.333333333	7.866666667	4.3	3.266666667
CEC2020_08	1.233333333	7.366666667	2.8	5	5.166666667	7.133333333	3.033333333	4.266666667
CEC2020_09	1.333333333	6.966666667	3.033333333	4.433333333	5.366666667	7.033333333	3.6	4.233333333
CEC2020_10	1.8	7.566666667	2.766666667	4.633333333	5.066666667	7.3	3.066666667	3.8
Avg Rank	1.585	6.5616	2.9183	4.5416	5.1883	6.8166	3.8416	4.5466
Final Rank	1	7	2	4	6	8	3	5

**Table 10:** Comparison of optimal solutions for the pressure vessel design problem.

Algorithm	Optimal Values for Variables				Cost
	T_s_	T_h_	R	L	
RCLSMAOA	0.742433	0.370196	40.31961	200	5734.9131
AOA [11]	0.8303737	0.4162057	42.75127	169.3454	6048.7844
SMA [15]	0.7931	0.3932	40.6711	196.2178	5994.1857
WOA [47]	0.8125	0.4375	42.0982699	176.638998	6059.741
GA [21]	0.8125	0.4375	42.0974	176.6541	6059.94634
GWO [48]	0.8125	0.4345	42.089181	176.758731	6051.5639
ACO [49]	0.8125	0.4375	42.103624	176.572656	6059.0888
AO [50]	1.054	0.182806	59.6219	39.805	5949.2258
MVO [51]	0.8125	0.4375	42.09074	176.7387	6060.8066

**Table 11:** Comparison of optimal solutions for the speed reducer design problem.

Algorithm	Optimal Values for Variables							Optimal Weight
	x_1_	x_2_	x_3_	x_4_	x_5_	x_6_	x_7_	
RCLSMAOA	3.4975	0.7	17	7.3	7.8	3.3500	5.285	2995.437365
AOA [11]	3.50384	0.7	17	7.3	7.72933	3.35649	5.2867	2997.9157
FA [52]	3.507495	0.7001	17	7.719674	8.080854	3.351512	5.287051	3010.137492
RSA [53]	3.50279	0.7	17	7.30812	7.74715	3.35067	5.28675	2996.5157
MFO [54]	3.497455	0.7	17	7.82775	7.712457	3.351787	5.286352	2998.94083
AAO [55]	3.499	0.6999	17	7.3	7.8	3.3502	5.2872	2996.783
HS [56]	3.520124	0.7	17	8.37	7.8	3.36697	5.288719	3029.002
WSA [57]	3.5	0.7	17	7.3	7.8	3.350215	5.286683	2996.348225
CS [58]	3.5015	0.7	17	7.605	7.8181	3.352	5.2875	3000.981

**Table 12:** Experimental results of three-bar truss design.

Algorithm	x_1_	x_2_	Best Weight
RCLSMAOA	0.78841544	0.408113094	263.8523464
MVO [51]	0.788603	0.408453	263.8958
RSA [53]	0.78873	0.40805	263.8928
GOA [59]	0.788898	0.40762	263.8959
CS [58]	0.78867	0.40902	263.9716

**Table 13:** Comparison of optimal solutions for the welded beam design problem.

Algorithm	Optimal Values for Variables				Best Weight
	*h*	*l*	*t*	*b*	
RCLSMAOA	0.20573	3.2530	9.0366	0.20572	1.6952
ROA [45]	0.200077	3.365754	9.011182	0.206893	1.706447
MGTOA [60]	0.205351	3.268419	9.069875	0.205621	1.701633939
MVO [51]	0.205463	3.473193	9.044502	0.205695	1.72645
WOA [47]	0.205396	3.484293	9.037426	0.206276	1.730499
MROA [9]	0.2062185	3.254893	9.020003	0.206489	1.699058
RO [61]	0.203687	3.528467	9.004233	0.207241	1.735344
BWO [62]	0.2059	3.2665	9.0229	0.2064	1.6997

**Table 14:** Experimental results of car crashworthiness design.

Algorithm	RCLSMAOA	ROA [45]	WOA [57]	MALO [63]	GTOA [64]	HHOCM [65]	ROLGWO [66]	MPA [67]
x_1_	0.5	0.5	0.8521	0.5	0.662833	0.500164	0.501255	0.5
x_2_	1.230638152	1.22942	1.2136	1.2281	1.217247	1.248612	1.245551	1.22823
x_3_	0.5	0.5	0.6604	0.5	0.734238	0.659558	0.500046	0.5
x_4_	1.198406418	1.21197	1.1156	1.2126	1.11266	1.098515	1.180254	1.2049
x_5_	0.5	0.5	0.5	0.5	0.613197	0.757989	0.500035	0.5
x_6_	1.08390407	1.37798	1.195	1.308	0.670197	0.767268	1.16588	1.2393
x_7_	0.5	0.50005	0.5898	0.5	0.615694	0.500055	0.500088	0.5
x_8_	0.345067013	0.34489	0.2711	0.3449	0.271734	0.343105	0.344895	0.34498
x_9_	0.347988173	0.19263	0.2769	0.2804	0.23194	0.192032	0.299583	0.192
x_10_	0.877748111	0.62239	4.3437	0.4242	0.174933	2.898805	3.59508	0.44035
x_11_	0.729351464	-	2.2352	4.6565	0.462294	-	2.29018	1.78504
Best Weight	23.18907104	23.23544	25.83657	23.2294	25.70607	24.48358	23.22243	23.19982

## Data Availability

Not applicable.
